# Paper-Based Humidity Sensors as Promising Flexible Devices: State of the Art: Part 1. General Consideration

**DOI:** 10.3390/nano13061110

**Published:** 2023-03-20

**Authors:** Ghenadii Korotcenkov

**Affiliations:** Department of Physics and Engineering, Moldova State University, MD-2009 Chisinau, Moldova; ghkoro@yahoo.com; Tel.: +373-60-642-109

**Keywords:** paper types, advantages, disadvantages, sensing materials, selection, comparison, sensor configuration, operation, fabrication, patterning, electrodes

## Abstract

In the first part of the review article “General considerations” we give information about conventional flexible platforms and consider the advantages and disadvantages of paper when used in humidity sensors, both as a substrate and as a humidity-sensitive material. This consideration shows that paper, especially nanopaper, is a very promising material for the development of low-cost flexible humidity sensors suitable for a wide range of applications. Various humidity-sensitive materials suitable for use in paper-based sensors are analyzed and the humidity-sensitive characteristics of paper and other humidity-sensitive materials are compared. Various configurations of humidity sensors that can be developed on the basis of paper are considered, and a description of the mechanisms of their operation is given. Next, we discuss the manufacturing features of paper-based humidity sensors. The main attention is paid to the consideration of such problems as patterning and electrode formation. It is shown that printing technologies are the most suitable for mass production of paper-based flexible humidity sensors. At the same time, these technologies are effective both in the formation of a humidity-sensitive layer and in the manufacture of electrodes.

## 1. Introduction

**Figure 1:**
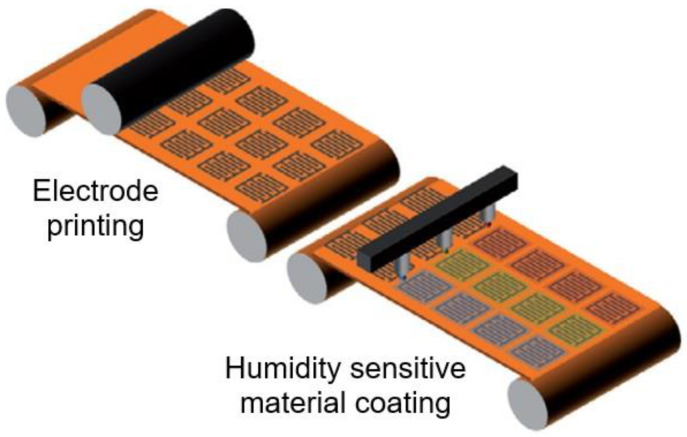
Schematic drawing of a roll-to-roll production line for chemical gas and humidity sensors on plastic foil. The transducers and coating layers are coated using additive printing techniques, such as the gravure printing of interdigitated electrodes and the local ink-jet printing of different sensing layers. Reprinted from [3]. Published 2011 by Elsevier as open access.

Lately, a new trend has appeared with the integration of sensors directly on flexible plastic foils. Their flexibility and their simplified processing, targeting the production on large area using roll to roll and printed electronics processing (see Figure 1), bring new opportunities and will also reduce several technical limitations that characterize the production processes of conventional microelectronics [1]. According to the Organic and Printed Electronics Association (OE-A) [2], in the coming years, printing technologies and printed electronics are expected to dominate in sectors such as the Internet of Things or IoT, healthcare, automotive and consumer electronics.

In conventional Si microelectronics, patterning is most often done using photolithography, in which the active material is deposited initially over the entire substrate area, and selected areas of it are removed by physical or chemical processes. Despite its high resolution, the photolithographic process is very complex, expensive, uses extremely expensive equipment, requires many steps, is time consuming, subtractive, and only suitable for patterning of small areas.

**Figure 2:**
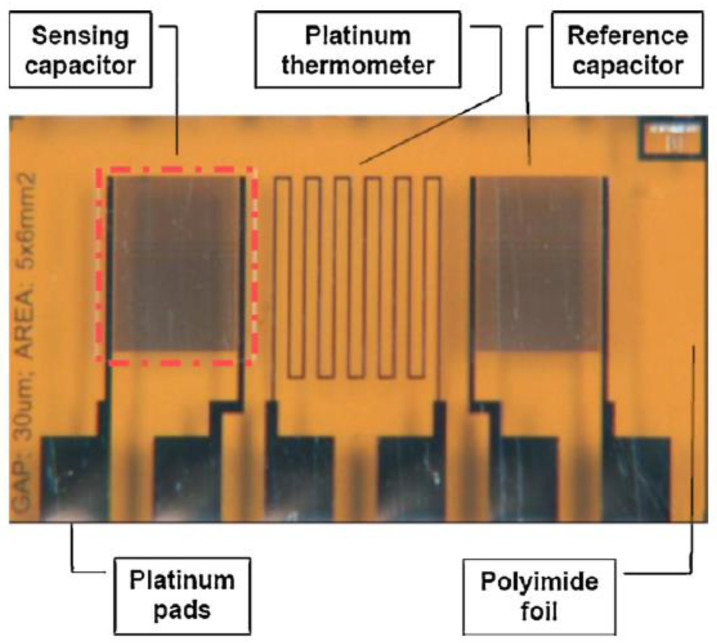
The sensor platform substrate with Pt thermometer, electrodes and connection pads. The interdigital electrode structures realize two plane capacitive transducers, a sensing one (**left**) and a reference one (**right**). The area reserved for the polymer sensing layer is surrounded by a dot line frame. Reprinted with permission from [13]. Copyright 2009: Elsevier.

In addition, the harsh conditions required to dissolve the resists, etch the underlying layers, and remove the photoresist destroy the activity of most organic electronic materials. De Rooij and co-workers [4,5] believe that the printing technology applied in flexible electronics is experiencing a significant growth and the sensors field can benefit from these developments with the availability of new types of materials and fabrication processes. Printed electronics can be defined as the combination of printing processes and ink chemistry for the manufacturing of electronic components. Compared to the conventional lithographic processes used in microelectronics, mentioned above approach to design of gas and humidity sensor as well to biosensors and electrochemical sensors guarantees reduced price, new functionalities and possibility to integrate sensors where it was impossible to imagine them a decade ago. This approach is compatible with the new generation of electronic devices made from polymeric materials (known as organic electronic devices), which are the future of lower manufacturing costs. This opens up a wide range of applications for flexible sensors such as environmental monitoring, food quality control, medicine and industrial process control [6,7,8,9,10,11,12]. An example of a flexible humidity sensor made on plastic foil is shown in Figure 2.

In addition, flexible substrates are ideal for deployment on curved surfaces, for integration on cloths for application in wearable sensors, smart textiles, or for development wireless radio frequency identification (RFID) tags for logistics applications [14]. In this case no direct optical contact is required to identify an item. It leads to higher efficiency in goods handling [15]. Plastic substrates possess also many attractive properties including biocompatibility, light weight, shock resistance, softness and transparency [16]. More detailed information on the achievements and prospects in the development of sensors on plastic foil can be read in the reviews prepared by Briand et al. [3] and Mattana and Briand [17].

Currently, a large number of flexible sensors have been developed. These include gas and humidity sensors, photodetectors, strain sensors, pressure sensors, temperature sensors and biosensors [18,19,20,21,22]. However, in this review, we will focus on the consideration of paper-based (PB) sensors [23,24,25,26], moreover, paper-based humidity sensors.

**Figure 3:**
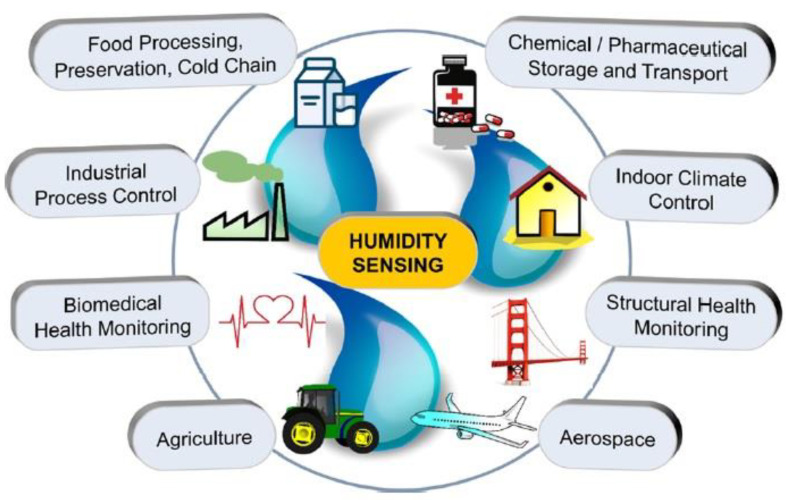
Various application paradigms of humidity sensors. Reprinted from [21]. Published 2021 by ACS as open access.

**Figure 4:**
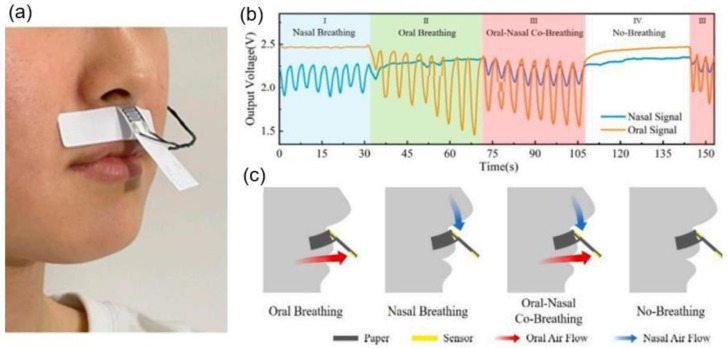
(**a**) Photo of a volunteer wearing a PB humidity sensor patch. (**b**) Simulated breathing experiments for different breathing modes and various respiratory patterns. (**c**) Schematic diagram of oral and nasal airflows in four respiratory patterns. PB humidity sensors was fabricated on printing paper. The graphite ink was screen-printed for fabrication interdigital electrode pattern. For the purpose of distinguishing between oral and nasal respiratory signals, two graphite screen electrodes were printed separately on both sides of the printing paper. Reprinted from [31]. Published 2022 by MDPI as open access.

Humidity sensors are among the fastest growing sensor markets. The global humidity sensor market is predicted to grow by 7.5% per year in the coming years and reach $1551.9 million by 2026 (https://www.researchdive.com/171/humidity-sensor-market, accessed on October 2022). This growth will primarily be due to the miniaturization of electronic devices and the use of humidity sensors in various industries. Due to unique water properties, humidity greatly affects living organisms, including humans and materials. The amount of water vapour in the air can affect the human comfort, and the efficiency and the safety of many manufacturing processes, including drying of products such as paint, paper, matches, fur, and leather; packaging and storage of different products such as tea, cereal, milk, and bakery items; and manufacturing of food products such as plywood, gum, abrasives, pharmaceutical powder, ceramics, printing materials, and tablets. Moreover, industries discussed above are only a small part of the industries where the humidity should be controlled. In agriculture, the measurement of humidity is important for the plantation protection (dew prevention), the soil moisture monitoring, and so on [27]. In the medical field, a humidity control should be used in respiratory equipment, sterilizers, incubators, pharmaceutical processing, and biological products. Humidity measurements on the Earth’s surface are also essential for meteorological analysis and forecasting, for climate studies, and for many special applications in hydrology, aeronautics, and environmental studies, since water vapor is a key factor in both weather and climate. Therefore, humidity control becomes mandatory in all areas of our activity, from production management to creating comfortable conditions for our living, and to understand the nature of climate change [23,24,25]. As a result, the field of application of humidity sensors is constantly expanding, requiring more and more devices with improved parameters and more adapted to new applications (Figure 3). The demand for paper-based flexible humidity sensors is caused precisely by these circumstances. In particular, in recent years, there has been a need for cheap flexible humidity sensors for applications such as breath analysis and respiration rate, diaper and skin moisture monitoring, healthcare monitoring systems, etc. [21,26,28,29,30]. Studies have shown that PB humidity sensors can be successfully used in these applications [26,28,31]. One example of such an application is shown in Figure 4. It is important to note that for such applications, humidity sensors, in addition to high sensitivity, good stability, long-term durability, rapid response and recovery time, high linearity, and exceptionally low hysteresis [18] must meet the following requirements [28]: (i) be flexible for wearables; (ii) not use toxic substances harmful to humans; (iii) be disposable to avoid cross-infection or contamination while monitoring the patient’s respiratory rate and wetting the baby diaper; (iv) have low cost and simple manufacturing technology; as well as (v) have a minimal polluting effect on the environment.

It should be noted that the field of paper-based sensors is developing rapidly, and significant progress has been made in the development of such sensors. At present, quite a lot of reviews on paper-based sensors have already been published [26,28,29,30,31,32,33,34,35,36,37,38,39,40,41,42,43,44,45,46,47,48,49,50,51,52,53,54,55,56,57]. But most of them are aimed at considering electrochemical and optical sensors, biosensors, gas sensors, and strain sensors. Humidity sensors have received less attention. At the same time, humidity sensors differ significantly from the sensors listed above in terms of the principles of operation, design, and sensitive materials used. Only a few review articles have attempted to review PB humidity sensors [26,28,29,31,49,57]. It has been shown that PB humidity sensors do indeed hold great promise for a wide range of applications. But in these articles, many aspects of the functioning, development, and manufacture of PB humidity sensors are either not considered, or this consideration was carried out without the necessary detail. This was the basis for preparing this review.

The review is organized as follows. It consists of two parts. In the first part of “General consideration” we give information about conventional flexible platforms and consider the advantages of paper for humidity sensor development as both a substrate and a humidity-sensitive material. Next, we discuss the manufacturing features of paper-based (PB) humidity sensors, their configuration, sensing materials and electrodes. Then, in the second part of this review “Humidity sensors performances”, we analyze the performance of paper-based humidity sensors manufactured using different approaches. Finally, we discuss the challenges encountered in the development and use of paper-based humidity sensors. Possible areas of application of PB humidity sensors are not considered in this article, since they are described in sufficient detail in the already published review articles mentioned earlier.

## 2. Conventional Flexible Platforms

One of the most fundamental challenges in the design of flexible electronic systems is the choice of substrate, because the material for such substrates must have a unique set of properties [58]. No doubts that substrates should be flexible. Specifically, this means that substrate must be able to bend but not crack or lose its other properties. Ideally, the substrate could repeatedly bend without a significant long-term degradation. Along with bending, it must be robust and cannot stretch. The substrate must withstand reasonable processing temperatures. This means that the melting temperature of the substrate must be sufficiently high. Additionally, the coefficient of thermal expansion (CTE) must be sufficiently low. If the film expanded or shrank (or both) too much under the heating, the layers deposited on the top (probably a mixture of inorganics that typically have low CTEs) are inclined to cracking or de-adhering just as in the case of external stress. The substrate should be thermally stable as well. Thermal stability is an important factor, primarily for fabrication reasons, because the ability to achieve very low processing temperatures is still the subject of investigation. The stability in aggressive environment is also required. In addition, a flexible substrate must be smooth and sufficiently adhesive, transparent, and above all, economically viable. There are a number of materials that meet most of these requirements and could possibly function as a flexible electronic substrate. However, the perfect material is not yet found and therefore at present time, when choosing a substrate, depending on its application, compromises are necessary.

At present, for substrates, flexible materials such as polyimide (PI), polydimethylsiloxane (PDMS), polyester (PE), polyethylene naphthalate (PEN), polytetraflouroethylene (PTFE), and polyethylene terephthalate (PET) are the most commonly reported ones [59,60,61,62]. PI is a widely preferred material as a substrate due to its chemical stability, radiation resistance, electrical insulation, and temperature stability. Polyimides maintain their properties during continuous use to temperatures from cryogenic to 232 °C and for short excursions, as high as 482 °C. As a result, polyimides are suitable for processing around 350 °C. Thermoset polyimides exhibit very low creep and high tensile strength. PET is another widely employed flexible substrate [63], owing to its good adhesion properties, low manufacturing cost, and commercial availability [64]. Depending on its processing and thermal history, PET may exist both as an amorphous (transparent) and as a semi-crystalline polymer. However, the glass transition temperature of these polymers is only in the range of 100–300 °C. Such low temperatures significantly limit the manufacturability of these films and require the development of new processing methods. PTFE allows for processing steps in the 250 °C range. PTFE has also excellent dielectric properties. Especially for conditions requiring tolerance to high temperature and stability of chemical properties, the PI substrate would be a better choice compared to PET and PTEF [65]. Meanwhile, Briand et al. [3] believe that polyimide will only be used for applications with specific requirements regarding temperature and the robustness of the substrate: most devices will be produced on PET and PEN substrates.

## 3. Paper as Flexible Substrate for Humidity Sensors

As follows from the previous discussion, the flexible organic materials play the irreplaceable roles in the substrates of flexible sensors because of their excellent flexibility and stability [66]. However, on the one hand, these organic substrates are difficult to degrade, resulting in electronic pollution [67]. For instance, plastics, such as PET (polyethylene terephthalate), are hazardous for the environment because plastic debris is a major source of marine pollution resulting in a rapid decline of global biodiversity [68]. On the other hand, the weak affinity between the sensing materials and organic substrates often leads to the detachment of the sensing materials during the deformation process, which greatly limits their durability [69]. Active materials, when coated on the top surface, cannot firmly adhere to the raw substrates, and even diminutive architectures can crack or tear during handling, bending, or stretching. Some developers note another disadvantage of polymer substrates, which is their limited active surface area [70]. The functional layers are formed on the surface of the substrate and therefore the intrinsic 3D architecture of materials such as paper cannot be exploited. Thus, the study of other flexible substrate materials is an important strategy for the development of high-performance flexible sensors. Paper has become one of the objects for such research [70,71].

### 3.1. Paper Types

Paper is a three-dimensional sheet formed by connecting cellulose fibers of different lengths through hydrogen bonds between hydroxyl groups. Paper is made from raw materials containing plant fibers through pulping, blending, and processing, and can be folded and cut as desired [72]. The cellulose fibers in paper are thinner than a micron but their length can be as much as tens of millimeters [73]. During the pulping process, various fillers such as pigments and chemical additives are added to give the paper different characteristics. For example, (i) adding mineral fillers such as calcium carbonate and clay to pulp can improve light scattering, ink absorption, and paper smoothness; (ii) by adding pulp such as starch, gum and rosin, the absorption of liquid by paper can be reduced and the strength of paper can be increased; (iii) addition of pigment coating can improve the smooth of surface and reduce pore size [70,74,75]. During the final processing, calendering and drying are used to form and dewater, and finally, the paper is given different appearances and sizes to achieve different applications [76]. The longer fibers that form the paper give it good strength, while the shorter fibers fill the gaps between the longer fibers, reducing pore size and making the paper opaque. In addition, cellulose fibers have a high aspect ratio, resulting in anisotropic properties [76]. By varying the length, diameter, and physicochemical properties of cellulose, paper properties such as porosity, density, and mechanical strength can be controlled [74].

The most common types of paper used in the development of humidity sensors are filter paper, nitrocellulose paper and office paper. Filter paper made from pure cellulose has various properties. Important parameters are wet strength, porosity, particle retention, volume flow, compatibility, efficiency and capacity. According to the quality of the filter paper, it is divided into qualitative filter paper and quantitative filter paper. Qualitative filter paper is often used in qualitative analysis. The reason is that qualitative filter paper produces more cotton fibers if it is used for filtering. Quantitative analysis filter paper is made in such a way that the paper does not react with common chemicals during the production process. As a result, it produces fewer impurities and is used in quantitative analysis. There are different grades of filter paper depending on the size of the pores. In total, there are 13 different grades of the filter paper. The largest pore size is grade 4; the smallest pore size—grade 602 h; the most commonly used grades are 1 to 4. Quality grade 1 filter paper has a pore size of 11 µm.

Nitrocellulose is a cellulosic compound produced by treating common cellulose with a sulphuric/nitric acid mixture, resulting in the substitution of (-OH) groups to (-NO_3_) groups in the polymer structure. Nitrocellulose surface is more homogeneous, smoother and has fewer and narrower pores in comparison to chromatographic cellulose-based paper surface [77].

Office paper can be understood to mean any commercially available commercial paper commonly used for office purposes. This category of paper-based substrates includes plain uncoated paper, coated photo paper (glossy or matte), card paper, and the like. As a rule, mineral fillers, such as calcium carbonate, chalk, and clays, are added to the paper base, usually in an amount of 10–20%, to fill the voids at the intersections of the fibers [78,79]. Additives such as starch, gum, and rosin can be added to paper to reduce liquid absorption and improve paper strength. To make the surface of the paper appear whiter and brighter, fluorescent bleaches (such as stilbenes) are often added during the papermaking process. The use of fillers improves the sheet shape, optical properties, printing properties of paper and reduces production costs. However, the gas permeability of the paper also decreases at the same time. It is the fillers and pigments that create a smooth and even surface with improved printing properties. Glossy paper is especially distinguished by these qualities. Thus, the type, shape, size and relative combination of filler and pigments are the main factors that determine the properties of office paper [80] and the final characteristics of paper as a platform for humidity sensors.

In addition to the above types of paper, it is also necessary to highlight the so-called nanopaper [71,81,82,83]. Nanopaper is a thin composed sheet mainly of densely packed nanostructures (such as nanocellulose, nanochitin, nanochitosan, nanographene, polymer nanofibers, carbon nanofibers, etc.). The most common is nanopaper based on nanocellulose. Three approaches are used to obtain nanocellulose: mechanical treatment, acid hydrolysis, and enzymatic hydrolysis [84]. The nanocellulose can be divided into two categories: cellulose nanocrystals (CNC) and cellulose microfibrils (MFC) also called cellulose nanofibrils (CNF) [82]. Nanopaper has significantly better physicochemical properties compared to ordinary paper [85,86,87]. Nanopapers made from the aforementioned nanomaterials are structured assemblies of nanomaterials that can bridge the gap between the nanoscale properties of such nanomaterials and their macroscopic engineering applications. Up-to-date, a number of nanopaper were developed in order to create products with additional functionalities including protected papers [88], low-gas permeable papers [89], transparent papers [90], superhydrophobic papers [91], fireretardant papers [92], antimicrobial papers [93], conductive papers [94], magnetic papers [95], sensor papers [96], shape-memory paper [97], and papers for energy harvesting and energy storage [98].

The experiment showed that a wide variety of paper types can be used to develop sensors [75]. The characteristics of some of them are listed in Table 1, Table 2 and Table 3.

### 3.2. Advantages and Limitations of Paper-Based Substrates

Paper-based sensors are considered to be a new alternative technology for making simple, low-cost and flexible electronic devices [26,70,71,74,105]. In particular, the unique properties of paper [106,107,108,109], such as its versatility, commercial availability, high quantity, low cost, small thickness, high porosity, adequate biocompatibility for bioassays, high thermal stability for robust applications, high mechanical strength to resist wear and tear, and elevated Young’s modulus values make also paper a promising sensor platform for the development of portable and disposable analytical devices for many applications, including clinical diagnostics, food quality control, and environmental monitoring [73,110,111]. For example, already in the early 20th century paper began to be used in chromatography [112], and in 1956 the first paper-based sensors were developed [113]. It was device for the semi-quantitative detection of glucose in urine. Since then, such detection devices have been further developed. Paper-based pregnancy tests were developed [114], and in 2007 a paper-based microfluidic device [115] was developed that could simultaneously detect both glucose and protein in artificial urine. Compared to the substrate materials of other microfluidic detection platforms, the paper used in these devices has the advantages of low cost, flexibility, and the ability to passively transport fluid due to capillary action [116]. In addition, the various properties of paper, such as thickness, porosity, roughness and wettability, make it possible to precisely regulate the microfluidic behavior to meet various requirements [117]. Cellulose fibers can also be chemically functionalized, in other words the properties of wood cellulose fibers such as hydrophilicity, permeability, reactivity, etc. can be tailored according to specific applications [110]. Comparative characteristics of paper and the most common substrates used in the development of humidity sensors are given in the Table 4.

During the research and use of paper, it was found that the three-dimensional porous structure throughout the thickness of the paper with a large surface area makes it easy to absorb functional reagents, and, most importantly, allows selective processing of two sides of the paper with different or the same functions [118]. In addition, cellulose, which is insoluble in classical organic solvents, gives paper excellent chemical stability [119]. Paper consisting of only cellulose also exhibits higher dimensional stability with temperature change and lower thermal expansion than most plastics [120], which is advantageous for electronic components since the use of paper does not introduce complex thermal parasitic effects into the behavior of electronic devices.

Paper as a dielectric material can be used in numerous sensing devices, especially capacitive-type sensors. In addition, the unique three-dimensional hierarchical architecture of cellulose fibers with different length scales allows the accommodate various active materials in substrates, opening up great opportunities for the development of advanced electronic devices. In this regard, in recent years, research has been carried out aimed at studying the possibilities of using paper substrates for the development of various sensors [121,122,123]. PB sensors such as gas sensors [26,29], temperature sensors [124], all-paper piezoresistive sensors for human motion detection [125], nanocellulose paper sensors for multichannel biological detection [126], pressure sensors [125], humidity sensors [26,29,127] and strain gauges [26] were developed. In addition, paper can be made thin, lightweight and flexible depending on its pulp processing. In addition, paper is combustible and biodegradable material, which greatly simplifies the recycling process [128]. It can be easily stored, transported and disposed [72]. Widely established as a recyclable material, paper products have a recovery rate of about 70 percent. According to a report by the United States Environmental Protection Agency on municipal solid waste (MSW), in the United States, paper waste constitutes 27.4% of the total MSW. However, the MSW recovery is dominated by paper at 51% [129]. The paper recycling process has significantly matured over the past decades. It saves tremendous amount of energy and reduces deforestation.

Moreover, cellulose fibres can be functionalized, thus changing properties such as hydrophilicity, if desired, as well as its permeability and reactivity [130]. The surface of the paper can be easily manipulated by changing the printing conditions, coating and impregnation. In addition, it can be produced in large quantities. Depending on the main goal to be achieved in paper-based sensors, the fabrication methods and the analysis techniques can be tuned to fulfill the needs of the end-user. For example, it has been reported that by filling inter-fiber space using transparent materials with reflective constant that is closed to the cellulose (≈1.5), it is able to fabricate transparent papers [74]. Reducing the diameter of cellulose fibers from micro-meters (≈20 μm) to nanometers (≈20 nm) will increase paper transparency [105].

Of course, paper substrates are inferior to plastic ones in terms of mechanical strength, resistance to aggressive environments, and manufacturability. The inherent surface roughness and porosity complicate the fabrication of devices on paper, especially when their size is reduced. However, there are paper processing methods that can significantly improve the properties of its surface [131]. For example, it has been shown that laser ablation [132] can be used to improve paper surface morphology and change surface energy, and plasma polymerization can be used to create hydrophobic polymer chains on the paper surface to make it water repellent [133]. Other interesting examples include the ability to improve the hydrophobicity of a paper surface by coating with organic or inorganic nanoparticles [134,135,136].

In addition, the large pore size in the paper results in poor thermal performance [137]. Moreover, cellulose itself is also prone to decomposition at temperatures above 100 °C [138,139,140]. Excess heating easily warps papers and degrades the quality of the cellulose structure. The upper limit of temperatures used for curing must be lower than 150 °C for a short period (such as 10 min). The degradation of cellulose results in a reduction in the mechanical strength of the paper [141]. This means that paper-based sensors are low temperature operated devices, and paper cannot be used in the high temperature processes often used to deposit humidity sensitive materials. This severely limits the types of deposition processes that can be applied to paper and the number of sensitive materials that can be used in PD humidity sensors.

Ongoing research also shows that paper substrates cannot replace plastic ones in all devices. But, in specific applications, paper substrates can undoubtedly find applications. For example, the high porosity of the paper makes it possible to incorporate materials that have properties that are important for sensor applications, but which are difficult to fix on plastic substrates. In addition, continuous pore channels, allowing for efficient diffusion of gaseous molecules throughout the film matrix, provide maximal exposure of sensing material to the gaseous analytes and thus makes it possible to enhance the sensor signal and accuracy [142].

Yao et al. [75], Hu et al. [143], and Singh et al. [144] believe that paper-based devices provide an inexpensive technology for fabrication of simple and portable diagnostic systems that can be immensely useful in resource-limited settings. The use of paper-based sensors will allow low-income regions to significantly expand the range of medical services provided at a low cost. Standard medical tests performed in centralized laboratories are either not available in such countries or are too expensive for most citizens. At the same time, paper-based sensors, inexpensive and easy to operate, could be used in resource-limited environments. Paper-based detection platforms also have great potential for use in remote areas and during emergency situations, where fully-equipped facilities and highly trained medical staff are absent.

### 3.3. Paper Selection

As we can see, there are a variety of paper materials available to the user. However, it cannot be said that paper of all types is a universal material suitable for all applications. Different types of paper have different properties and therefore the choice is based mainly on the fabrication steps required in developing a device and also on the specific application area [108,110,145,146]. In particular, the filter paper (the Whatman^®^ cellulose range, Maidstone, UK) is most suitable for developing microfluidic sensors due to its wicking ability [147]. The range of Whatman^®^ filter papers is popular due to the choice of paper with the required porosity (pore size of 11–25 µm) to control particle retention and flow rates [148,149]. The difference between paper grades lies on the coarseness and packing of the cellulose fibres. Different grades allow different sampling and assay settings to be applied. Due to the porosity of Whatman^®^ filter papers, flow rate, and particle retention, this paper is suitable for fabricating electrochemical devices capable of storing the reagents, filtering the sample, enabling reactions to occur, and flowing the detectable product towards the electrochemical testing area [150]. Paper towel also has properties similar to filter paper. Paper towel is cheaper than filter paper and possesses a high porosity, which makes it a viable material for analysis of a wide range of analytes [151,152].

Nitrocellulose, obtained from the partial nitration of cellulose, enhances the porous property of cellulose and changes the material from hydrophilic to hydrophobic due to the presence of nitro groups. It has smaller pores than those of Whatman Grade 1 paper. Nitrocellulose membranes exhibit a high degree of non-specific binding towards biomolecules and are suitable for immobilization of enzymes, proteins and DNA [122,153]. In addition, nitrocellulose membranes are smooth and have a reasonably uniform pore size, which results in a more stable and reproducible liquid flow within the paper. This makes hydrophobic nitrocellulose suitable for the development of various biosensors. Bioactive paper has also been used in biosensors. In the bioactive paper, the paper matrix is modified with biomolecules. This facilitates the adsorption of biomolecules. One of the challenges of using nitrocellulose is related to the difficulty of working with this paper because it is fragile and difficult to handle. In addition, the oxidation of nitro groups is observed [109]. These shortcomings have significantly limited the use of this paper.

We must not also forget about office paper. The structure of office paper can be used in various configurations. For example, due to the non-degradability and relatively smooth surface of glossy paper, it is a good substitute for filter paper especially when modifying nanomaterials onto a surface rather than within the fibre matrix is necessary [154]. In addition, due to the lower porosity of the office paper, electrodes can be formed directly on the surface without penetrating deep into the paper. Moreover, the ordinary A4 printing paper has been found to be the most widely used for making PB flexible humidity sensors due to its good mechanical flexibility, rough and porous structure, good hydrophilicity, and easy availability [26]. Santhiago et al. [155] showed that it is possible to develop wearable devices based on office paper, which should be foldable and flexible. Chromatographic filter paper has high fragility after wetting

Conventional paper is known to have disadvantages such as optical opacity, high surface roughness, and low mechanical strength and low stability in aqueous media, which may hinder further development of paper-based sensor devices for special applications [122,156]. On the other hand, it has been found that nanopaper, mainly made from densely packed renewable natural nanomaterials, not only has many of the advantages of conventional paper, but also eliminates many of its disadvantages, offering much higher transparency, better chemical, thermal and mechanical stability, much lower thermal expansion and lower surface roughness [156,157]. Besides, nanopaper maintains its structure in the aqueous media, compared with conventional paper. This makes nanopaper an excellent platform for the production of high-performance paper-based sensors [158]. The potential application of nanopapers for humidity sensing was reported by Li et al. [159]. Giese et al. [160] developed cellulose nanopapers undergo rapid and reversible changes in color upon swelling, which show potential applications in humidity sensing. Nogi et al. [161] used cellulose nanopapers with a high surface smoothness as a substrate for the antenna to transmit and receive signals, which is an important component of a satellite, computer, or other radio-frequency identification (RFID)-based devices. As for the disadvantages of cellulose nanopaper, they usually include the following [162]: (i) poor retention in fibrous materials; (ii) relatively high cost; (iii) negative effect on paper drainage and drying, (iv) increased tear susceptibility, and (v) high energy consumption in production. This is what limits the widespread use of cellulose microfibers (CNFs)- and cellulose nanocrystals (CNCs)-based substrates for the development of cheap flexible humidity sensors.

In addition to the examples above, unusual paper substrates such as carbon fiber paper (CFP) and paper hybrids can be found in paper-based sensor designs. Carbon fiber paper is a composite paper composed of carbon fibers and polymeric carbon. Its production process begins with polymerization and carbon fiber formation, followed by carbonization, papermaking, resin impregnation, molding, and a final heat treatment step [163]. CFP has been primarily explored as a building material for electrodes, due to its exceptional conductivity, high chemical stability, and good mechanical properties. Limitations of CFP include low electrochemical activity and small surface area [164]. It has been reported that cardboard can also be used as an alternative to paper. Its use was first proposed in [165], where laser pyrolysis of organic compounds present in cardboard formed a highly ordered graphite-like structure used as a conductive electrode.

A discussion of the advantages of certain types of paper for the development of flexible devices can be found in published reviews. In particular, Liu et al. [71] summarized the diverse paper substrate options and fabrication techniques for PB electronics. Barhoum et al. [83] and Naghdi et al. [158] reviewed the flexible and multifunctional nano-papers and analyzed their possible applications. Zhang et al. [70] reviewed the micro/nanostructured papers as substrates for flexible electronics. Mahadeva et al. [166] considered paper as platform for energy and sensing applications.

As follows from this consideration, the mechanical, chemical and electrophysical properties of each paper type differ from each other due to differences in the material composition and manufacturing processes. As a result, it is hard to generalize a set of properties for paper as the constituents of paper vary among different types of paper [72]. For example, printing paper has some cellulose fiber with a large amount of filler material. The filler material can either be natural materials (limestone, clay, and talc) or synthetic alternatives (precipitated calcium carbonate, titanium dioxide, and gypsum). The quantity and type of filler materials define the structure, thickness, and appearance of the paper [167]. The filler dictates the cost of production, refractive index, paper strength, brightness, energy required for drying, friction, pore size, and burn rate of the paper [168,169,170]. Fillers can negatively affect the strength, retention, abrasion, dusting, and sheet two-sidedness. Thus, the diversity in the types and quantity of filler material used in each type of paper is what makes the properties of paper (in general) so diverse. This has remained a challenge for researchers to theoretically identify the properties of the paper which they used in their research.

## 4. Configuration of Paper-Based Humidity Sensors and Mechanisms of Their Operation: General Consideration

In terms of device architecture, the humidity sensor comprises a sensing/transduction layer over a substrate with electrodes placed for physical interfacing. As a rule, paper-based humidity sensors are developed in two modifications that differ in their sensing mechanisms. These are capacitive and resistive. There are also impedance-based humidity sensors, but they are much less common. Other humidity sensors that show responses at different frequencies like quartz crystal microbalance (QCM) [171], surface acoustic wave (SAW) and Lamb wave types have been reported [172], but they are usually constructed from rigid platforms.

The most common way to manufacture paper-based (PB) humidity sensors is to apply a humidity-sensitive material to the surface of a paper substrate. It is assumed that the paper in such a sensor is a passive element, and the sensor performances are controlled by humidity-sensitive material. It is clear that not every material can play the role of humidity-sensitive material in paper-based humidity sensors.

### 4.1. Sensing Materials

According to [23,24,25,173], the ideal characteristics of a humidity-sensitive material are such properties as high sensitivity to water vapor, i.e., excellent affinity for water molecules and large active surface area, low cross sensitivity to other gases in the atmosphere, long service life, fast and reversible interaction with analytes, no long drift, efficient low cost technology, high reproducibility and good adhesion, i.e., uniform and strong bonding to the surface of the substrate. Experiment has shown that various types of sensing materials, including carbon materials and their derivatives (carbon nanotubes (CNTs), graphene oxide (GO), reduced graphene oxide (rGO)) [174,175,176,177,178], metal oxide semiconductors [179,180], organic materials [181,182], metal sulfides [183], and others [184], can be used for fabricating PB humidity sensors.

Carbon materials have been extensively studied as humidity-sensitive materials for flexible devices due to their high surface area, low toxicity, high electron mobility, mechanical compliance and good chemical stability [185]. Graphene is currently the most studied carbon-based material. But, it has an important drawback. Its deposition requires high temperatures, which are unacceptable in the manufacture of paper-based sensors. As a result, graphene is typically deposited on a rigid substrate prior to being transferred to a flexible substrate [186,187], which makes it difficult to manufacture cheap sensors. At the same time, graphene oxide (GO) is a derivative of graphene that offers the possibility of room temperature deposition using techniques as simple as coating and self-assembly. In addition, unlike graphene and reduced GO (RGO), GO is strongly hydrophilic and proton conductive, which makes it a superior material for water vapor sensing like the typical proton conducting material. Therefore, it is graphene oxides that are commonly used in the development of humidity sensors [177]. The sensing mechanism between a carbon-based material (CNTs and GO) and water molecules depends on the presence of hydrophilic functional groups on its surface such as hydroxyl groups. Graphene oxide is naturally functionalized with hydrophilic groups such as hydroxyl (-OH) and epoxy (=O) [188]. Upon its exposure to humidity, water molecules bind with the hydroxyl (-OH) and epoxy (=O) functional groups with binding energies of 0.201 and 0.259 eV respectively, thus withdrawing electrons from the graphene oxide [189]. As a result, graphene oxide becomes less conductive when exposed to a reducing gas such as water. Thus, the GO’s hydrophilic functional groups (hydroxyl and epoxy) enhance the adsorption and desorption of water molecules and thus enhance the sensitivity of the material. Numerous active sites, such as defects, and vacancies are also able to capture water molecules and provide a sensory response to the appearance of moisture.

The common advantages of sulfide-based nanomaterials like MoS_2_, VS_2_, WS_2_, ZnIn_2_S_4_, and CdS in humidity sensing are their rich hydrophilic surface sites, which allow the occurrence of proton transition induced by the absorbed water molecules as long as the humidity level is increased. Additionally, due to the ultrathin 2D layered semiconductors, it is possible to create transparent humidity sensors.

Like metal sulfides, nanomaterials based on metal oxides also have hydrophilic properties necessary for use in humidity sensors [190]. Nanomaterials based on metal oxides with excellent electronic properties are mainly used for flexible resistive or impedance humidity sensors [191].

Polymers is another group of materials widely used in the manufacture of humidity sensors [192]. Polymers are distinguished by low cost, simple synthesis, high stability, high transparency, superelasticity, outstanding mechanical properties, and the presence of various functional groups, such as –COOH, –OH, and –NO_2_ [193]. The humidity sensitive properties of polymers are mainly determined by the hydrophilic characteristics of functional groups, which can form rich hydrogen bonds with water molecules [194].

Humidity sensing properties of materials used for humidity sensor fabrication are discussed in sufficient detail in [23,24,25,195,196,197,198]. Humidity has been found to affect material properties through various interactions such as hydrogen bonding [199], intramolecular contacts [200], electrostatic interactions [201], hydrophilic and hydrophobic interactions [202], chemical bonding [203], etc. As previously stated, different materials are sensitive to humidity of the air. However, the conventional metal oxides gas sensing materials that usually require high temperature for synthesis are difficult to be used for fabricating PB humidity sensors because the paper substrate is not resistant to high temperature [26]. From metal oxides, only materials that can be synthesized at low temperature can be used in PB humidity sensors. This ability, in particular, has ZnO [204].

As it was indicated before, the paper begins to degrade at temperatures above 100 °C, placing severe limitations on the quality of crystalline semiconductors that can be grown directly on the paper. Therefore, in many cases, developers prefer to use a polymer and carbon-based materials as sensing material. However, it is known that polymer- and carbon-based humidity sensors have limitations in many applications. For resolving above problem with deposition of crystalline material, three various approaches for overcoming temperature restriction have been proposed [205]. The first approaches are crystallization methods, in which an inorganic semiconductor is found that can be deposited onto paper, and subsequently crystallized at low temperatures. An example is the conversion of amorphous material into polycrystalline one via laser crystallization. The second method is the wet-transfer, or ‘bottom-up’ methods. Single-crystalline material is prepared at high temperatures, then synthesized material is transferred onto the paper at ambient temperatures in the form of solution or paste and then it is exposed to low temperature treatment, admissible for substrate used [206]. The local ink-jet printing is also a very promising method for the preparation of different sensing layers [207]. The third approach is dry-transfer method, involving the relocation of semiconductor materials or fully fabricated devices from inorganic substrates to the paper, using poly(dimethylsiloxane) (PDMS) stamps or soluble glues. We need to note that all mentioned above methods can be used for the manufacture of humidity sensors.

The roughness and porosity of the papers tend to hamper the performance of some electronic devices such as transistors and diodes; on the contrary, the roughness and porosity are attractive here because they increase the contact area with the ambient air and promote the adhesion to sensing materials used. Similar to the methods of forming sensitive material on polymer flexible substrates, the main methods of applying sensitive materials used in the fabrication of PB humidity sensors include handwriting [176,180,208], spin-coating [209], vacuum filtration [178,210], drop-casting [175,182], printing [211,212,213] and soaking treatment [182]. Due to the rough, porous, and hydrophilic surface of paper, the handwriting and vacuum filtering are relatively unique to PB sensors compared to ceramic and organic substrates. At that, in the manufacture of PB humidity sensors, high-temperature methods of direct growth of high-quality thin-film sensitive materials on a paper substrate, such as chemical vapor deposition (CVD), are generally not applicable. When fabricating PB humidity sensors, the method of printing sensitive materials should be preferred. Meanwhile, the rough and porous surface of the paper also creates problems in the manufacture of PB sensors using this technology. This technology requires high printability of sensitive materials (e.g., size of the particles, heat resistance, dispersion, and surface energy). Among these fabrication methods, handwriting (painting, drawing) and drop-casting are often used due to their simple and inexpensive processes [26].

It is clear that when designing flexible humidity sensors, developers very often fail to strike a balance between humidity sensitivity and response/recovery rate due to inherent properties of the materials used. Therefore, various strategies have been developed to optimize performances of humidity sensors, such as chemical treatment, alloying, transition to nanocomposites, structural engineering, Joule heating, etc. [190,214,215,216,217].

### 4.2. Sorption Isotherms

**Figure 5:**
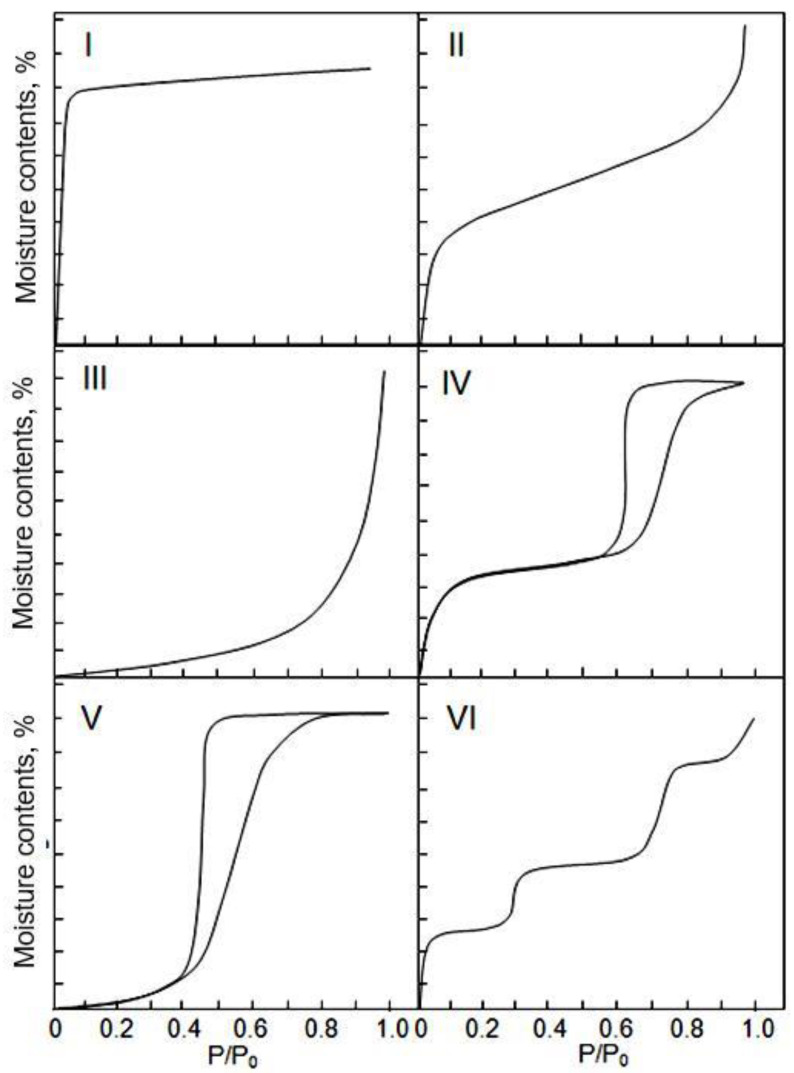
Basic shapes (**I**–**VI**) of sorption isotherms according to Brunauer S. Adapted from [219]. Published 1985 by IUPAC as open access.

The main process that determines the sensory response to moisture is the adsorption of water vapor on the surface of the active material. Adsorption processes of water vapour taking place in porous media usually is described by an adsorption isotherm [218,219,220,221]. Although, in the literature many types of isotherms for different absorbents and adsorbed media can be found, most of them can be classified into one of the five or six isotherms classes originally formulated by Brunauer [220]. The original types of isotherms are presented in Figure 5. Type I is characteristic for purely microporous adsorbents such as zeolites or activated carbon, type II for nonporous or purely macroporous materials, type III for the rare cases of nonporous adsorbents with very small interactions between the adsorbent and the adsorbed medium. Type IV isotherm characterizes mesoporous adsorbents and is very common, contrary to the type V which presents a relatively rare case of mesoporous adsorbents with small interactions with the adsorbed medium. Type VI is very rare; the adsorption process is realized here in several separated steps. As we can see, an adsorption isotherms are non-linear. This means that capacitive humidity sensors—as well as other absorption-based humidity sensors, typically show a non-linear behavior as a function of relative humidity (RH).

**Figure 6:**
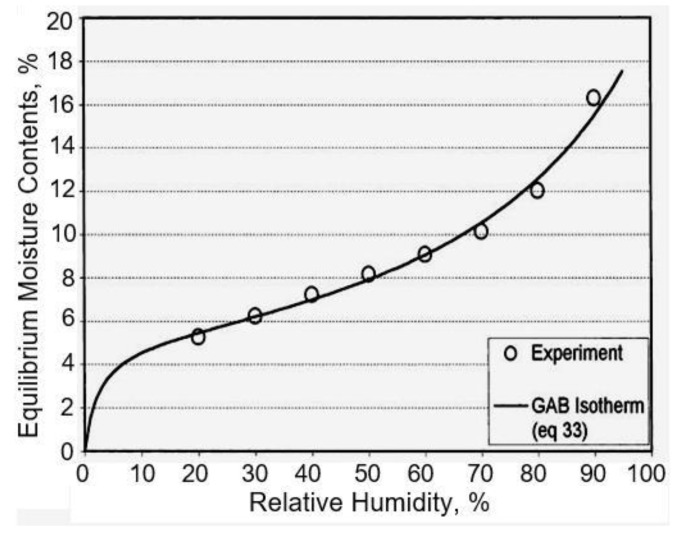
Sorption isotherm of bleached kraft paperboard (BKP) at 23.9 °C and atmospheric pressure. Reprinted with permission from [224]. Copyright 2003: ACS.

In porous materials used in humidity sensors, such as paper, the adsorption isotherms are almost exclusively of type II, i.e., with an S-shape [222,223]. Typical adsorption isotherm of water vapor on the surface of the paper is shown in Figure 6. At low relative humidity, the molecules of water are bound in one layer to the surface of pores by van der Waals forces. This phase of adsorption expresses the relatively fast moisture increase in the initial regions of the curve. The molecules of the first adsorbed layer have a character similar to the liquid phase. Before the end of the adsorption in the first layer, further layers appear. This process is characteristic for a plane surface of the adsorbent and bigger pores where the curvature of the adsorbed film does not affect the gas pressure of the adsorbent above it. This phase is characterized by the linear part of the isotherm. The final phase is the water vapour condensation.

It is important to take into account that at desorption, we sometimes get a different dependence of the water content on the relative humidity than at adsorption [221]. The desorption isotherm always lies above the adsorption isotherm. This effect is called hysteresis. When the humidity sensor is operating, this effect is manifested in the fact that the sensor readings, depending on the direction of the change in humidity, in the direction of increasing or decreasing, may differ. Hysteresis is typical for isotherms of types IV and V (Figure 5), but it is often observed for isotherms of type II. Hysteresis is caused by two main mechanisms. First, this is the hysteresis of the contact angle, which is much smaller during adsorption than during desorption. Probable reasons for this fact are water contamination of a solid surface, surface roughness, or immobility of the surface water layer [225]. The second mechanism is related to the delayed desorption of water vapor from nanopores. Theoretically, hysteresis can also be caused by a third factor, namely, irreversible processes occurring during water adsorption, but it is assumed that in most cases this factor is much less significant than the two previous ones [226].

### 4.3. Paper as Humidity Sensing Material

**Figure 7:**
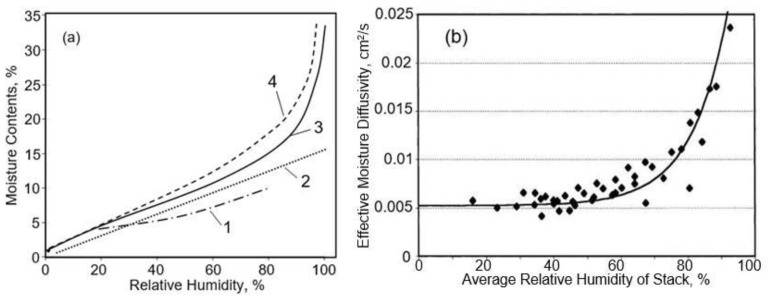
(**a**) Moisture content in 160 g/m^2^ paper in dependence on RH. 1—Data extracted from [234], 2—[235]; 3—[236]; 4—[237]; (**b**) Effective moisture diffusivity of bleached kraft paperboard (BKP) at 23.9 °C and atmospheric pressure. Adapted with permission from [224]. Copyright 2003: ACS.

It is important to note that the properties of paper make it possible to use it not only as a substrate in the development of humidity sensors, but also as an active humidity sensitive material. The experiment showed that the paper has a hydrophilic surface. In brief, cellulose paper has a large amount of hydrophilic –OH groups that can adsorb water molecules. At low relative humidity, only a small amount of water molecules is absorbed by -OH groups via hydrogen bonds, resulting in a slight increase in paper conductivity [26]. At high relative humidity, a large number of physisorption water molecules are adsorbed on the paper surface, which contributes to the formation of a larger number of conductive ions and an increase in ionic conductivity of the paper. For instance, at a relative humidity (RH) of 70–80%, paper absorbs up to 10–15% of its weight in water and up to 35 w% at 100%RH (see Figure 7a). Since the moisture content of the paper in the investigated RH range (0–90%) was linearly dependent on RH (Figure 7a), the resistivity of the papers is exponentially dependent on the moisture content. The exponential dependence of resistivity on paper moisture content is well known, but different mathematical expressions for this dependence have been proposed [227,228]. In most cases, the dependence of surface and volume resistivity of the paper on RH is described by expression:

*R* = *A*e^−RH/*B*^, (1)

where *A* is the resistivity at zero moisture content and *B* is a factor which reflects the sensitivity of the paper resistivity to the moisture content or to the RH at which the paper was conditioned. Since the ionic conductivity of the paper is proportional to the amount of water adsorbed on the surface of the cellulose fibers, then such properties of paper made it possible to use it as a humidity-sensitive material for the manufacture of PB humidity sensors [28,229,230,231,232,233].

Studies have shown that humidity sensitive properties of the paper can be improved by surface and bulk modification and functionalization [108,166]. For example, the salt content in the paper changes the conductivity through hygroscopic effects. Results demonstrating this effect have been presented in [238]. It was also established that ionic addition not only increases the number of available free ions but also changes the water state and the paper structure, which influence the ionic motion in an electric field [234]. At that O’Sullivan [239] established an interesting regularity in the effect of salt content on the conductivity of paper. He concluded that the conductivity of cellulose (cellophane, which is hydrated cellulose) containing 1% or more salt is determined primarily by the moisture content, but when salt content is clearly below this limit, salt content also becomes an important factor for the level of conductivity.

As mentioned earlier, the dielectric properties of paper also change when water vapor is adsorbed [46], which makes it possible to design paper-based not only resistive ones [209], but also capacitive ones [232] and impedance humidity sensors [240]. It is important to note that the use of paper in its natural form as a humidity-sensitive material is indeed an important advantage of paper in the development of inexpensive and easy-to-manufacture moisture sensors, since in this case the paper in the humidity sensors simultaneously plays the role of both substrate and sensitive material. It should be taken into account that paper is not only a cheap alternative to other humidity-sensitive materials, but also provides high sensitivity and speed due to its porous structure.

With regard to moisture transport in the paper, as a result of the studies carried out, it was concluded (see Table 5) that moisture can migrate in paper by a number of transport mechanisms, such as (i) vapor-phase diffusion in the inter-fiber pore space, (ii) Knudsen diffusion in pores of diameters less than 10 nm, (iii) surface diffusion over fiber surfaces, (iv) bulk-solid diffusion within fibers, and (v) capillary transport [241,242]. The first two mechanisms occur in the gas phase, whereas the other ones occur in the condensed state of the liquid (adsorbed, absorbed, and liquid water, respectively). Liang et al. [241] found gas-phase transport to be the dominant mode of moisture movement, whereas condensed-phase or bound-water movement could occur when the moisture content (MC) was high. Nilsson et al. [242] concluded from their experimental work that moisture transport in paper occurred via gas-phase diffusion at RH levels below 58% rather than via the transport of absorbed water molecules. However, according to an earlier study of Ahlen [243], the transport of water in the condensed or bound state could be significant even at RH levels as low as 30–40%. Hashemi et al. [244] observed the in-plane diffusivity of moisture in paper to be a strong function of its MC. Gupta and Chatterjee [224] have also shown that the moisture diffusivity of paper strongly increases with moisture (see Figure 7b). A more detailed analysis of the processes that control moisture migration in paper can be found in [224,245,246].

**Figure 8:**
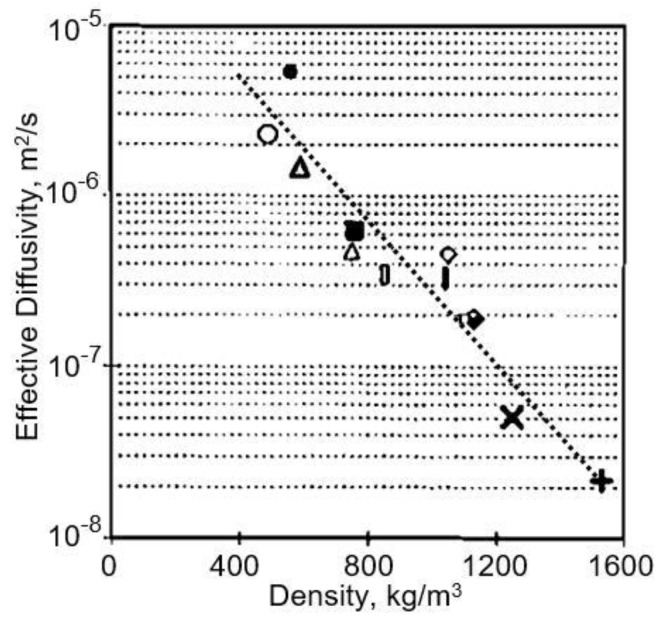
Effective water vapour diffusivity as a function of the density of the paper sheet. Reprinted with permission from [242]. Copyright 1993: Taylor and Francis.

One of the results of the conducted research is the establishment of a direct correspondence between water vapor diffisivity and the paper density. The effective water vapor diffisivities measured for different types of paper are shown in Figure 8. It is seen that the diffisivities range in a wide interval from 2.1 × 10^−8^ m^2^/s to 5.4 × 10^−6^ m^2^/s, which means that the diffusion of water vapour through the paper/pulp grades is reduced by between 5 and 1400 times compared with the diffusion of water vapour in air. The highest value for effective vapour diffisivity was found in the filter paper, and the lowest value in the lightweight coated (LWC) paper, which had been coated and calendered.

### 4.4. Capacitive Paper-Based Humidity Sensors

#### 4.4.1. Mechanism of Sensitivity

Capacitive sensors are the most commonly used humidity sensors and have the advantage of being quick and simple to react to humidity. Capacitive sensors are linear and can operate over a wide humidity range. In addition, capacitive sensors are less sensitive to bending strain and temperature. In the simplest case, a capacitive-type sensor consists of two parallel plates. In such a structure, the capacitance between two electrodes is given by the Equation (2):

(2) $$C=\varepsilon_{r}\varepsilon_{0}\frac{A}{d}$$

where ε*_r_* and ε_0_ are the relative and vacuum permittivity constants, respectively, *A* is the plate surface area, and *d* is the plate distance. It can be seen from this equation that there are only three ways to change the capacitance of this device: (1) change the distance *d* between the two plates, (2) change the overlap area A between the two plates, and (3) change the dielectric permittivity of the material between the plates. This means that capacitive sensors can detect only those gases and vapours that affect these parameters. Water vapour can exert such influence and therefore by measuring the change in the capacitance, the presence of the water vapours in the air can be detected. In particular, if we take into account that the dielectric constant of water is 80, and paper, depending on the type, lies in the range of 2–4, it will be clear that the adsorption of water can lead to a significant change in the dielectric constant of the paper. In comparison, polymers have a dielectric constant of 3–6, metal oxides have a dielectric constant of 3.6–9, and carbon-based materials have a dielectric constant that varies between 6–15. It is clear that the lower the dielectric constant of the dielectric used and the greater the proportion of the space between the electrodes is occupied by water, the greater will be the effect, which is manifested in increasing the capacitance. In other words, high adsorption capacity and high porosity, which can be filled with water, are important parameters for materials suitable for the development of capacitive humidity sensors.

It is important to note that for a noticeable change in capacitance, water in a dielectric material must be in a free state, since only free water molecules have dielectric properties close to those of liquid water, while bound water exhibits dielectric properties characteristic of ice. According to Evans [247] and Matzler and Wegmuller [248], fresh water ice has a permittivity of 3.17–3.19, which is significantly less than that of water. Unfortunately, water condensation on the surface of a solid, i.e., the appearance of fresh water occurs at 100% humidity. However, in the presence of pores in the material, especially in the nanoscale range, the situation changes dramatically. According to the basic theory of adsorption on a porous matrix [249], when the vapor molecules are first physicosorbed onto the porous material, capillary condensation will occur even at low air humidity if the pores are narrow enough. The critical size of pores for a capillary condensation effect is characterized by the Kelvin radius (Equation (3)). In the case of water, the condensation of vapour into the pores can be expressed by a simplified Kelvin equation [250]:

(3) $$r_{K}=\frac{2\gamma V_{M}cos\theta }{\rho RTln(\%RH/100)}$$

where *γ* is the surface tension of vapour in the liquid phase, *V_M_* is molecular volume, *θ* is contact angle, and *ρ* is the density of vapour in the liquid phase. Equation (3) was obtained for case of cylindrical pores [251]. Thus, the condensation occurs in all pores with radii up to *r_K_*, the Kelvin radius, and under a constant water vapour pressure or relative humidity. The smaller *r_K_* or the lower the temperature, the easier capillary condensation occurs, i.e., at lower air humidity, free water is formed in the pores of the dielectric.

Considering that condensed water has the maximum effect on the capacitance, it becomes obvious that it is the pore size and porosity of the humidity-sensitive material that determine the sensitivity of the capacitive humidity sensor. The smaller the pore size is, the lower is the sensitivity threshold, i.e., at a lower humidity level, capillary condensation and an increase in capacity begin. At that, the greater the porosity and open pores volume are, the greater is the amount of condensed water and the greater is the range of capacity change when interacting with water vapour. It is important to note that capillary condensation can occur in all humidity sensitive materials used in the manufacture of humidity sensors, which by definition must be porous. These include paper, which has a porous structure with pore sizes varied from 0.7 nm to 8 µm. However, it should be borne in mind that the reduction of pore diameter will influence simultaneously the kinetics and hysteresis of sensor response, i.e., the response time and the magnitude of hysteresis will increase.

**Figure 9:**
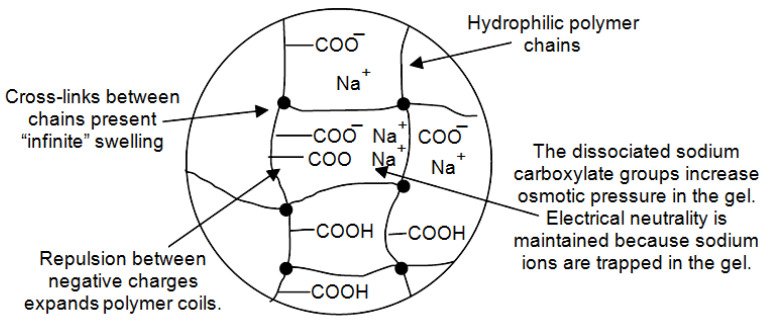
Diagrammatic representation of the part of the polymer network. Adapted with permission from [253]. Copyright 1997: Wiley.

**Figure 10:**
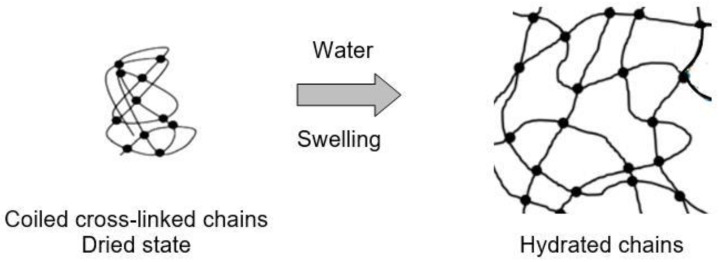
Changes in a three-dimensional network of polymer chains during swelling.

A change in capacitance associated with a change in the distance between the electrodes is also possible if the interaction with water vapor is accompanied by a swelling effect. This effect in cellulose paper was observed by Olejnik et al. [252]. However, the swelling effect is most pronounced in the interaction of water vapor with polymers. There are several mechanisms to the process of swelling, which include hydration and the formation of hydrogen bonds [253,254,255]. Schematically, they are shown in Figure 9 and Figure 10.

In reality there is a balance between the forces of retraction and the tendency for the chains to swell to infinite dilution. The degree of cross-linking has a direct effect on the level of swelling of the polymer and the strength of the network, i.e., increased cross-link density = decreased swelling capacity. This means that the swelling effect has strong dependence on the technology of polymer synthesis. Regarding a swelling rate, experimental studies have shown that for a rapid effect, the polymer should be a macro-porous (the large pores with size in the range of 0.1–1 μm), or even super-porous, i.e., along with high porosity, polymer should have interconnected open-cell structure [256]. In this case the swelling is very fast and this effect is sample size-independent.

One should note that, the position and the shape of an adsorption isotherm, shown in previous section, strongly depend on the temperature [221]. At higher temperatures, the transport of water molecules is faster, the bonds can be released more easily, and therefore both adsorption and desorption isotherms corresponding to higher temperatures are lower (or shifted to the right) as compared to those corresponding to lower temperatures (some water molecules already bound on the solid surface can be released, polymolecular layers on the pore walls are thinner). Therefore, the capillary condensation occurs (all other conditions being the same) at higher relative humidity. All this leads to the fact that the readings of capacitive humidity sensors, like other humidity sensors, are temperature-dependent.

#### 4.4.2. Configuration of Capacitive Humidity Sensors

**Figure 11:**
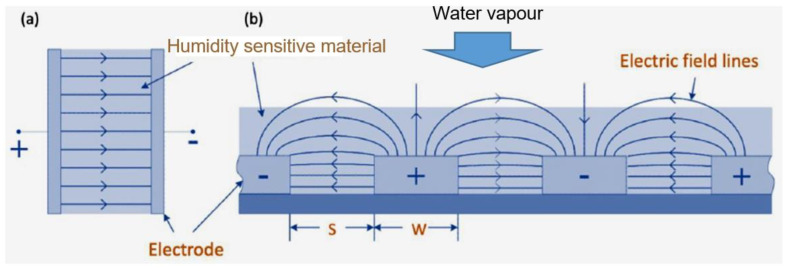
(**a**) Parallel plate capacitor; (**b**) coplanar interdigital sensors and electric field distribution in them. Adapted from [257]. Published 2015 by MDPI as open access.

**Figure 12:**
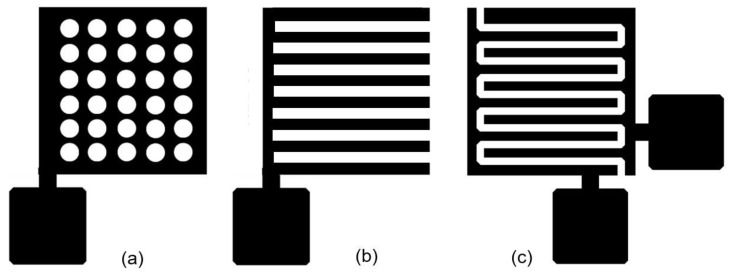
Possible configuration of electrodes in capacitive humidity sensors: (**a**,**b**)—perforated electrodes in parallel plate capacitive humidity sensors, (**c**) interdigital electrodes in coplanar capacitive humidity sensors. Adapted from [229]. Published 2014 by MDPI open access.

In classical capacitive sensors a humidity sensitive material is placed between two, the top and bottom, electrodes (see Figure 11a). This is so called the parallel plate structure. The sensor material is made very thin to achieve a large signal change with humidity. In order to ensure the access of water vapour to the humidity sensitive material, the upper electrode must be a water vapour permeable, i.e., porous. This also allows water to easily exit the sensitive material, allowing for quick drying and easy sensor calibration. As a rule, inert conductive materials, stable in the presence of water vapour, are used to form these electrodes. The simplest solution is the deposition of a thin film of gold or platinum, which at a thickness of 10–20 nm has sufficient water molecules permeability. In the case of using paper characterized by high porosity and surface roughness, this thickness should be increased. Metal film perforation can also be used. In addition, this approach provides the best reproducibility of the parameters. There are a huge number of such options. Two of them are shown in Figure 12a,b. An intermediate solution is to provide the top electrode with a fine mesh.

However, the experiment showed that when using the configuration of a plane-parallel capacitor, certain technological difficulties arise for the implementation of PB humidity sensors with better sensor characteristics. To achieve the maximum capacitive response to humidity and reduce the effect of parasitic capacitance, the thickness of the sensitive layer should be minimal, and the area of the upper electrode occupied by the conductive material should be maximum, since this area determines the capacitance value. A minimum sensing layer thickness is also required for fast response. But the requirement for fast response contradicts the requirement for increasing the area of the electrodes. This is due to the fact that the diffusion of water vapor under the top electrodes is in most cases the phenomenon that determines the response rate. If water diffuses rapidly into the thin sensing layer from the surface to the bottom electrode, then in metal plated areas water diffuses sideways under the top electrodes, and this diffusion rate can be quite low. In other words, sensitivity and response time are inversely related: if the structure is optimized for faster response, then the sensitivity will decrease, and vice versa. Therefore, the choice of the optimal structure is a compromise between these requirements.

As a result of the research, we came to the conclusion that the configuration of planar sensor, when both electrodes are in the same plane (Figure 11b), is better suited for the development of capacitive humidity sensors. In this case, the electrodes can be formed both on the surface of the sensitive layer and preliminarily on the surface of the paper substrate before applying the sensitive layer. Various configurations of such sensors have been tried [258,259], but the most optimal option was of coplanar interdigital (ID) sensor (see Figure 12c). The term “interdigital” refers to a digit-like or finger-like periodic pattern of parallel in-plane electrodes [260] that used to build up the capacitance associated with the electrical fields that penetrate into a material sample. These designs, allowing for a uniform electrical distribution in the dielectric and give the possibility for the water vapor to diffuse freely into the dielectric (see Figure 11b). Experience has demonstrated that ID electrodes provide the optimal balance of sensitivity and manufacturing yield [24]. The planar interdigital sensor works on the same idea as a two parallel plate capacitor, with electrodes that open up to enable one-sided contact with the sensing material. The electrode separation specifies the width of the empty area between consecutive electrodes [259].

As a result of testing the capacitance-type IDE sensors, it was concluded that achieving the maximum signal requires a certain ratio between the thickness of the sensitive material and the IDE geometry, since the electrodes’ periodicity and the thickness of the sensitive coating layer impact the response of a capacitance-type IDE sensor [183,261]. In particular, it was observed that the electrodes should have the largest feasible thickness and the smallest possible width for optimal effectiveness [24].

### 4.5. Resistive Humidity Sensors

The humidity measurement in resistive sensors is based on the change in resistance or conductivity of a humidity-sensitive film produced by contact of humidity sensitive material with water vapor. The presence of water vapor modifies the electrical conductivity (or conductance) of the sensing layer by phenomena such as adsorption, chemical reactions, diffusion, and swelling (in the case of polymers) that occur on the surface or in the bulk of the sensing layer. This modulation may be quantified as a change in current, resistance or conductivity that are proportional to the water vapor concentration in the surrounding environment [24].

**Figure 13:**
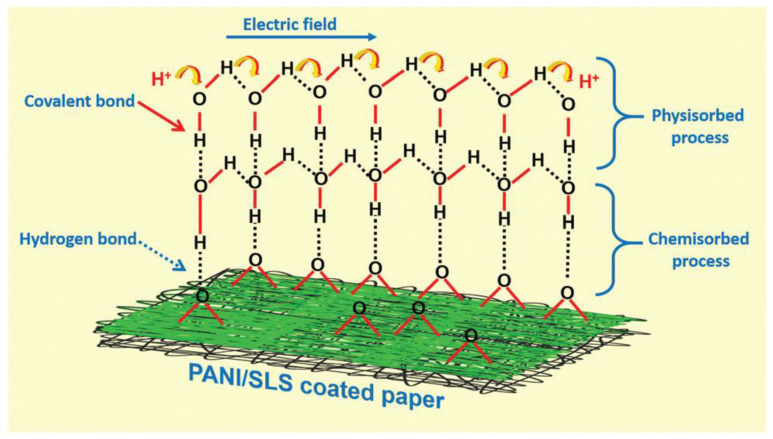
Schematic diagram depicting the sensing mechanism of the polyaniline (PANI)/SLS-X coated paper-based humidity sensor. Reprinted from [263]. Published 2022 by RSC as open access.

In principle, the basic mechanism of conductivity in the majority flexible humidity sensors can be explained by the Grotthuss reaction, which is usually simplified as a proton-hopping process [262]. It usually refers to the dynamic process of charge transfer within humidity sensitive materials in a humid atmosphere. Since the signal intensity of a humidity sensor reflects the number of water molecules adsorbed on the surface of sensitive materials, the hydrophilic properties of active materials play a vital role in terms of sensitivity performance. As a rule, the process of absorption of water molecules can be divided into two stages (see Figure 13). First, the first layer of water molecules is adsorbed on the surface of active materials by chemical bonding with hydroxyl groups or surface defects. As RH increases, more layers of water molecules are formed on the surface of sensitive layer through physical adsorption. Since water molecules are easily ionized in an electrostatic field, hydroxyl and proton ions are spontaneously generated and transferred between neighboring water molecules. This greatly facilitates the transfer of charge carries and thereby changes the output of the humidity sensors.

Measurements in resistive sensors are usually carried out with a constant voltage applied to the electrodes. It is important to note that, just like in capacitive humidity sensors, the ID electrode configuration is the most optimal when manufacturing resistive humidity sensors.

### 4.6. Impedance Humidity Sensors

Impedance humidity sensors are a type of devices that operate on the basis of a change in impedance. A sinusoidal voltage is applied to the humidity sensor in the frequency domain from subhertz to megahertz, and the impedance is calculated by measuring the current. The advantage of this type of humidity sensors is that they can accurately detect relatively low humidity. But, as a rule, this requires professional high-performance impedance spectroscopy. Despite the relative complexity of the measurement process, studies have shown that this method not only allows one to estimate the level of humidity, but also provides useful information about the mechanism of interaction of water vapor with the material [264].

Typically, impedance-based humidity sensors are configured identically to resistive sensors. Since measurements in impedance-based humidity sensors are carried out on alternating current, detectable changes in impedance are the result of changes in both capacitance and resistance of the sensing element [240]. Generally, the impedance decreases gradually as the operating frequency increases. The impedance curve tends to flatten out at higher frequencies, indicating that the impedance is independent of relative humidity in this frequency range [181]. This is due to the fact that the polarization of water molecules is difficult to keep up with the rapid change in electric fields at a higher frequency. Therefore, it is best to measure humidity at a relatively low frequency.

**Figure 14:**
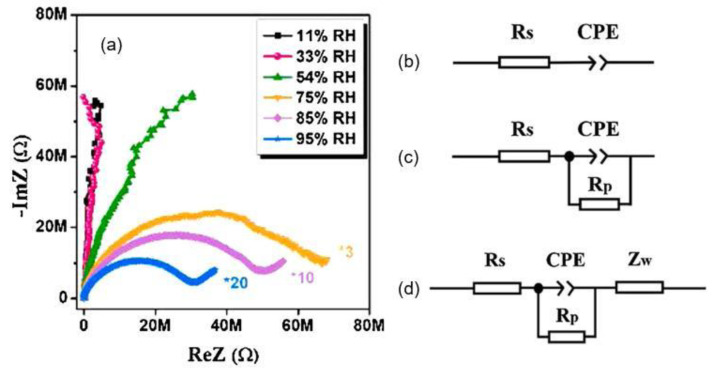
(**a**) The complex impedance spectroscopy plots of blank paper at different RHs. (**b**–**d**) The corresponding equivalent circuits. CPE- constant phase element; R_p_- equivalent film resistance; Z_w_- Warburg impedance. Reprinted with permission from [217]. Copyright 2021: Elsevier.

Typical the complex impedance plots (CIPs) of paper-based humidity sensor without additional humidity-sensing element is shown in Figure 14a. In low humidity environment, the impedance plot presents an arc shape with a large radius of curvature, which indicated that the sensor operates as a capacitance-type device (Figure 14b). As the RH increases to 75%RH, the Rez-Imz curve becomes a complete semicircle, and the equivalent circuit (EC) of the sensing film is consistent with that of a parallel capacitor and resistor (Figure 14c). The appearance of the resistance branch indicates that a conducting path forms inside the sensing film (Figure 13d). This occurs when the hydrophilic cellulose fibers adsorb a large number of water molecules by chemical adsorption and form a discontinuous layer of water.

## 5. Features of Fabrication of Paper-Based Humidity Sensors

Paper is fundamentally different from the substrates used in the production of classical microelectronic devices. Therefore, the technologies used in conventional Si technologies are not applicable or have significant limitations for use in the manufacture of paper-based detector. This means that the development of paper-based devices requires new approaches and new technologies.

### 5.1. Patterning

Given the scope of paper devices, in their manufacture, it is necessary to search for technologies and materials that meet the criteria of low cost, simplicity and efficiency of the production process [265,266]. Techniques used for patterning reported in the literature include photolithography, analogue plotting, inkjet printing and etching, plasma treatment, paper cutting, wax printing, flexography printing, and screen printing [75,108,122,265,266]. Experiment has shown that laser treatment can also be applied for this purpose. In particular, de Aranjo et al. [165] reported the first example involving the use of a single-step CO_2_ laser scribing process to pattern nanostructured electrodes on paper, providing a green solution for reagentless mass-scale production.

With respect to photolithography commonly used in microelectronics, there is a possibility of damage to the photoresist when paper is bent or folded when photolithography is used to make paper-based sensors. To overcome the problem associated with photolithography, Bruzewicz et al. [267] proposed the use of an elastomeric polydimethylsiloxane applied to paper using a plotter. This allowed the paper device to be rolled up without destroying the photoresist. In addition, this method is highly reproducible and uses inexpensive materials, and thus is suitable for even the most basic of research laboratories where sensors with an element size of ~1 mm are being developed. The advantages and disadvantages of technologies implemented in the manufacture of paper-based sensors are listed in the Table 6. As you can see, all methods have both advantages and disadvantages [75,144], and therefore, patterning methods must be chosen depending on the type of material used and the type of modification required [268]. If the stripe resolution needs to be in the millimeter range, screen printing or pencil drawing may be the best choice. However, while the former allows mass production with satisfactory reproducibility, pencil drawing is more suitable for proof-of-concept than for scalable processes. If micrometric strips are required, inkjet printing offers a great advantage given that patterns can be drawn (and redrawn) with common software. Of course, photolithography and plasma processing provide the highest resolution. However, the requirements for photoresists, solvents, and special equipment, make them less suitable for ‘real’ cost-effective fabrication of humidity sensors [268].

As for the description of patterning technologies listed in Table 6 and suitable for the manufacture of PB humidity sensors, they are described in detail in many review articles [25,75,144,269], and therefore will not be considered in this review.

### 5.2. Electrodes

Electrodes play an important role in humidity sensors, especially those of the resistive type. The resistance of the electrodes should be as low as possible. Therefore, for their manufacture it is necessary to use highly conductive materials. For example, Arena et al. [154] used integrating multi-walled carbon nanotube electrodes. Steffens et al. [270] developed a low-cost sensor using graphite interdigitated electrodes. A simple and scalable method to fabricate graphene-cellulose paper was reported by Weng et al. [271]. Carbon based materials have become a popular choice in electrode fabrication for sensors due to their low cost, high conductivity and availability. Conductive materials are also used, such as metal nanoparticles (Ag, Au, Pt, Pd, etc.). However, the most common among them in the manufacture of humidity sensors are still silver electrodes [64,151,272]. Copper has also been of interest owing to the high conductivity of bulk copper and the lower price compared to silver. However, instability of the copper particles in environmental conditions is a major drawback to the use of copper in conductive metallic inks, and several researches have focused on the protection of copper particles [273].

Metallic particle inks offer the highest level of conductivity. To obtain a high level of conductivity, an additional post treatment of annealing (~100–250 °C for several minutes) is usually required. This leads to the formation of a continuous interconnected phase between the metallic particles after the elimination of all the insulating components present in the ink (stabilizing agents, other additives, etc.). Silver is the most widely used metal so far owing to its high bulk conductivity and high resistance to oxidation. As a result, Ag nanoparticle inks have excellent low electrical resistance, when they are printed and cured on paper substrates. Most of the conductive silver inks are based on spherical nanoparticles or flakes. These inks exhibit a high conductivity but the obtained printed tracks and layers are usually opaque and brittle.

**Figure 15:**
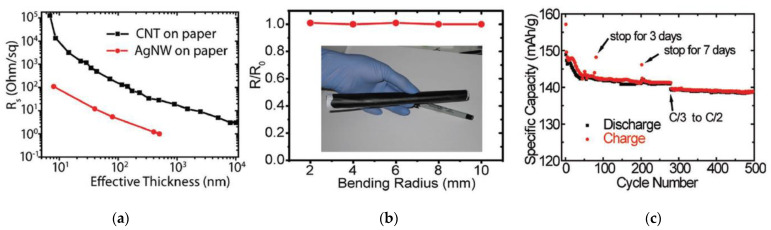
(**a**–**c**) Electrical performance of conducting paper based on CNTs and Ag. Reprinted from [69]. Published 2009 by National Academy of Sciences as open access.

Carbon-based materials such as carbon nanotubes (CNTs), graphite and graphene are another group of materials successfully used to make electrodes on the surface of paper. For example, Hu et al. [69] fabricated a highly effective conducting paper electrode with robust chemical and mechanical stability using the simple conformal coating of CNTs-ink onto commercial photocopy paper. However, experiment has shown that for practical applications, the use of composites based on carbon-based materials is more promising. Hu et al. [274] fabricated a highly conducting porous composite of the nanocellulose fibers and CNTs for application as high-performance electrodes by simply uniformly mixing of the two components. Hu et al. [69] have also shown that due to the continuous electrical conduction pathways formed by combining CNTs with silver nanowire, the composite CNTs-Ag NWs allows the formation of electrodes with very good electrical parameters and mechanical flexibility (see Figure 15). The sheet resistance of the electrodes increased only slightly (<5%) even after bending to a 2 mm radius 100 times. Hu et al. [69] believe that this inspiring result is likely ascribed to the joint impact of the flexibility of individual CNTs, the strong binding between the CNTs and the cellulose, and the porous architecture of the cellulose paper, which can greatly mitigate the applied bending strain. Additionally, the strong attraction of the cellulose fiber to the CNTs provided high film stability against damage (e.g., scratching and peeling off). The conductive performance of flexible graphene-cellulose paper electrode is also acceptable, and only 6% decrease was found after 1000 repeated bending tests [271].

In some publications, conductive polymers such as poly(3,4-ethylenedioxythiophene) (PEDOT) [275] or polyaniline (PANI) [276] are also used to form conductive layer and electrodes. The conductivity, flexibility and transparency of these materials are different [82]. Conductive polymer and carbon particles inks differ from metal inks as they possess a moderate conductivity (about 100 times lower as compared to silver), but can exhibit transparency and flexibility. These inks do not require any annealing treatment, allowing the printing on flexible substrates such as plastics, which cannot withstand high temperatures [277,278]. Some details about specific inkjet conductive materials are discussed in [82,279].

**Figure 16:**
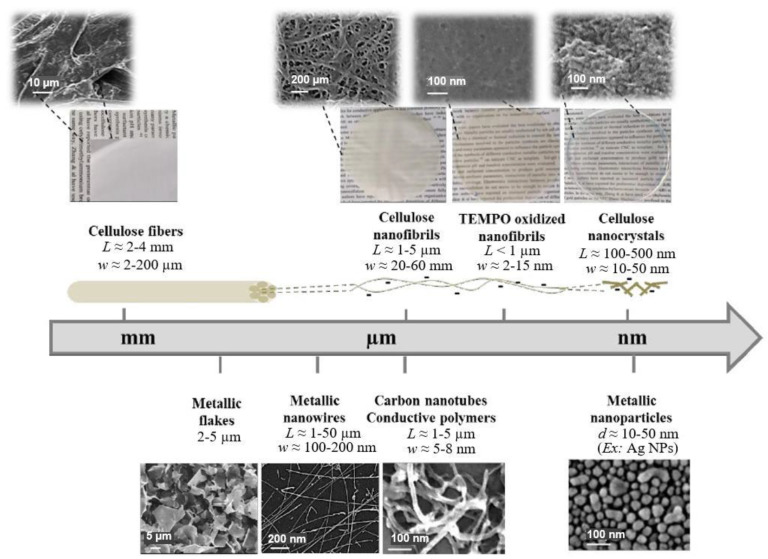
Schematic length-scale representation of networks obtained with different cellulosic materials (**up**) compared to specific dimensions of different conductive particles (**down**). Reprinted with permission from [82]. Copyright 2016: RSC.

Figure 16 summarizes the conductive particles and their length scales as compared to nanocellulose. The conductive particles are generally selected by (i) the printing process due to specific physico-chemical ink properties and particles dimensions and (ii) the required performance for end-use application in terms of flexibility, transparency and conductivity.

**Figure 17:**
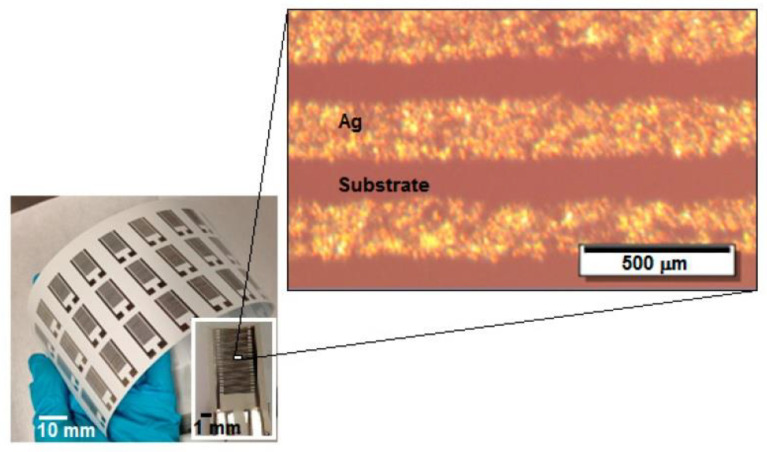
Digital picture of an array of inkjet-printed Ag interdigitated finger electrode structure and the magnified view of one device and the electrodes (5X magnification). The printed structures showed average width of ~208 µm, a gap between fingers of ~140 µm, and average thickness of ~185 nm. Silver (Ag) nanoparticle colloidal ink (ANP 40LT15C) was used. Reprinted from [232]. Published 2017 by MDPI as open access.

As with conventional substrates, a variety of methods can be used to fabricate paper-based electrodes [280]. For example, various printing technologies can be used [212,269,281] and magnetron sputtering [282]. In particular, Nee et al. [147] and Dungchai et al. [283] used screen-printed electrodes on paper for electrochemical analysis. Cinti et al. [151] used inkjet printing technology to form carbon black electrodes. Inkjet printing technology has also been used by Gasper et al. [232] for the formation of silver interdigitated electrodes in the production of capacitive humidity sensors (see Figure 17). Hu et al. [284] used inkjet printing to fabricate arrays of nanoporous gold electrodes on cellulose membranes. An advantage of inkjet-printed electrodes is that electrode thickness can be tuned by printing multiple layers to lower resistance and improve robustness [285]. Inkjet printing also eliminates some of the waste involved in screen and stencil printing and provides better resolution, which may become important for intricate patterning. Ink is deposited in the desired areas, thus removing the need of using masks, and the thickness of the deposited materials can be precisely controlled [275]. Due to the already automated nature of this technique and reduced waste, inkjet printing electrodes would be able to move to large-scale production efficiently. Gaspar et al. [232] noted also that usually in the process of applying ink to the surface of the paper substrate, some of the ink immediately saturates the paper fibers and penetrates into the paper. This not only promotes the drying process by increasing the evaporation rate of the ink solvent, but also improves adhesion. At the same time, it improves the high resolution of the print by avoiding bleeding on the surface of the substrate, improving the definition of edge line roughness.

The limitations of inkjet printing are especially due to the specific ink physicochemical properties requirements for allowing droplet ejection and the restriction in the particle size (<1 μm) for avoiding nozzle clogging [286]. In these technologies, a wide range of carbon and silver inks can be used to produce electrodes [147,272,283]. In particular, commercial CCI-300 Ag-ink (Cabot, Inc.) having an Ag nanoparticle diameter in the range of 30~50 nm in ethylene glycol/ethanol as the main solvent can be used to form electrodes. You can also use silver nanoparticle ink (DGP 40LT-15C, Sigma-Aldrich, Sigma-Aldrich, MO, USA). Au, Cu and Pt nanoparticle inks can also be used to form electrodes [279,287]. Yao et al. [288] found that in order to produce high-quality electrodes using printing technology on an office paper substrate, pre-treatment of the paper is necessary to remove inactive additives and increase its porosity. They suggested the following procedure: immerse a sheet of printing paper in an aqueous solution containing 0.3 M hydrochloric acid (HCl) for about 10 min, then rinse thoroughly with deionized water and allow to dry at room temperature. Pretreatment typically results in a reduction in both weight and thickness due to the removal of mineral fillers and fluorescent bleaching agents, resulting in a more porous open structure, which is often useful for paper-backed electrodes. Courbat et al. [289] for improving the quality of the electrodes, before printing with Ag-ink, the paper was dehydrated at 110 °C for 2 h, without using any surface pretreatment.

It was also found that in the manufacture of silver contacts, post-treatment drying at room temperature of about 25 °C followed by treatment at T ~ 150 °C is necessary to achieve maximum conductivity. At that only less than 2 min of drying time at this temperature was required to achieve the best electrical conductivity [290]. It was also found that photon annealing may be a better option in the manufacture of Ag-based electrodes [291]. When using photon sintering methods, the effect of nanoparticle displacement during annealing is practically absent. The photon energy is more localized and efficient, which means that solvent evaporation and nanoparticle coalescence occur at almost the same rate, preventing nanoparticles from moving to the edges of the printed lines, providing a more uniform electrode thickness. Another common effect when using thermal sintering is crack formation, or microcracking [292], resulting in disruptive and non-conductive structures. Paper has a low thermal expansion when compared to polymers, and that is the main reason why the crack formation due to substrate shrinkage is almost non-existent [293]. It was also found that electrodes made with other materials also require heat treatment at 60–90 °C for several minutes after printing. It was also found that electrodes made with other materials also require heat treatment at 60–90 °C for several minutes after printing.

Experiment has shown that besides surface pre-treatment and post-deposition thermal treatment [294,295], there are many additional factors that must be clarified when using paper substrates for preparing electrodes using printing technology. Wetting, spreading or permeability behavior of nanoparticles inks is quite different from plastic substrates. It is necessary to control the spreading or permeation of the inks. Another concern is the interface reaction or the compatibility of the papers with the ink during curing. The papers must maintain its structure while forming a strong interface during curing.

Hand painting is also a frequently used electrode preparation method. Liu et al. [71] drew conductive traces with high conductivity, remarkable foldability and stability on paper by using prepared AgNW-GO ink and a ballpoint pen, and verified the performance of the electrode. It is found that graphene oxide (GO) can work as the dispersing, associate thickening, stabilizing, adhesive, oxidation-resistant, and mechanical reinforcing agents simultaneously to formulate a drawable conductive ink with silver nanowire (AgNW). Liu et al. [71] believe that this method can be used to quickly prepare electrodes without any expensive equipment. But major disadvantages of this approach include the influence of the painter, low repeatability and the inability to draw on a large scale.

**Figure 18:**
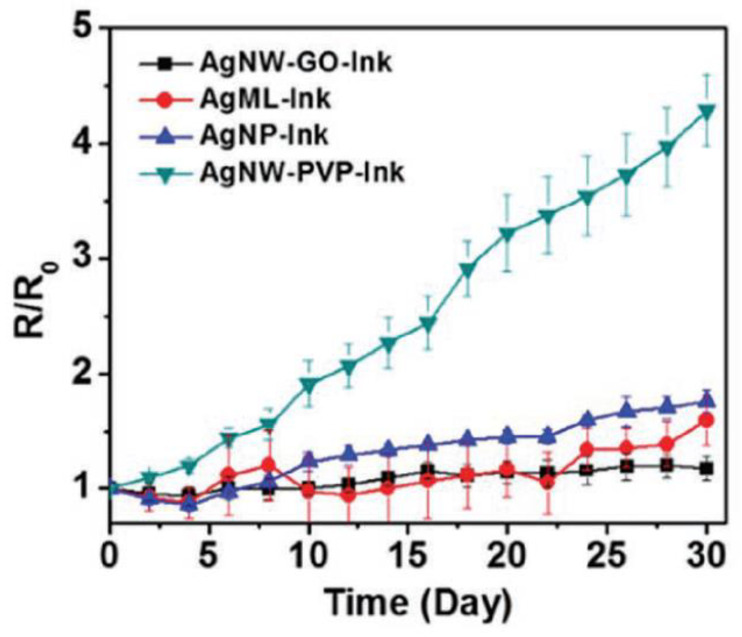
Plots of relative resistance change versus time for AgNW-GO-Ink, AgNW:PVP-Ink, AgNP-Ink, and AgMF-Ink electrodes drawn on paper substrates after exposure to hot air at 60 °C for 30 d. AgMF- silver microflakes; AgNP—silver nanoparticles, AgNW—silver nanowires. Reprinted with permission from [71]. Copyright 2017: Elsevier.

With regard to the stability of Ag-based contacts, studies by Liu et al. [71] showed that electrodes made using AgNW-GO ink not only have low resistance, but also have increased stability compared to electrodes based on AgNW-PVP, AgNP, and AgMF inks. As it is seen in Figure 18, after accelerated test at 60 °C in air over 30 days, the resistance of AgNP-Ink and AgMF-Ink electrodes has a tendency of slight increase over testing time and reaches to 1.8 and 1.6 times their initial values. However, the resistance for AgNW-PVP-Ink electrode exhibits a much greater increase and rises to 4.3 times its initial values after 30 days. It was established that the resistance increase is mainly attributed to surface oxidation issue of the nanoscale or microscale silver fillers caused by the attack from environmental oxygen and sulfur [296,297]. In contrast, the resistance of the AgNW-GO-Ink electrode increased by a mere ≈15% after 30 d. It is suggested that the GO wrapping around the AgNWs and their junctions can work as a barrier layer to impede the AgNWs from reacting with environmental oxygen and sulfur [296,297,298]. Such long-term storage stability is important advantage of AgNW-GO electrodes for application in paper-based electronics. Molina-Lopez et al. [64] found that the stability of Ag-based electrodes could be improved by passivation of printed silver electrodes with nickel.

**Figure 19:**
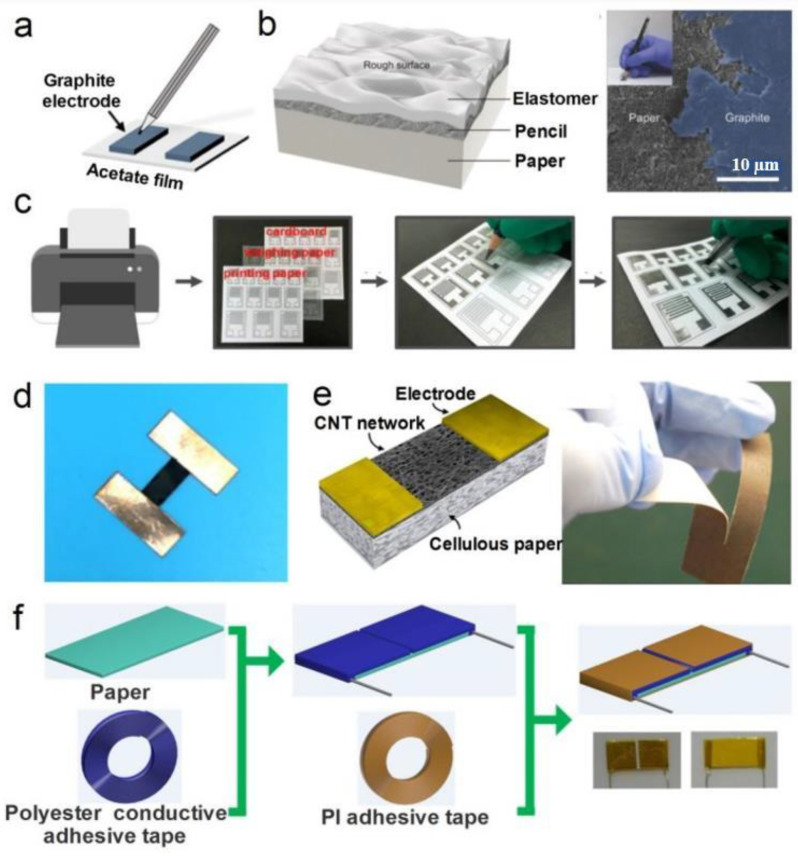
(**a**) Fabrication schematic illustration of the pencil electrodes. Reprinted with permission from [180]. Copyright 2018: American Chemical Society; (**b**) Schematic illustration of the microstructure-like randomly rough surface. Reprinted with permission from [301]. Copyright 2017: Wiley-VCH; (**c**) Fabrication schematic illustration of the PB electrodes using drawing method. Reprinted with permission from [176]. Copyright 2017: American Chemical Society; (**d**) Photograph of the PB humidity sensor based on copper electrodes. Reprinted with permission from [178]. Copyright 2019: American Chemical Society; (**e**) Schematic illustration of the humidity sensor built on a cellulose paper substrate using copper electrodes. Reprinted with permission from [175]. Copyright 2012: American Chemical Society; (**f**) Fabrication process of the PB humidity sensor using pasting method. Reprinted with permission from [28]. Copyright 2019: American Chemical Society. (Reprinted with permission from [26]. Copyright 2020: ACS).

Benefiting from the rough surface of the paper, the electrodes of PB sensors can be prepared by simple, low-cost and solvent-free processing methods such as pencil drawing [176,180,181,299,300]. Conductive graphite is the main component of the pencil, and this is what allows the use of a pencil drawing for the manufacture of PB electronic devices [26]. Figure 19a–c shows the pencil drawing electrodes for PB sensors. It was established that the sheet resistances of the PB electrodes fabricated by different types of pencil are different due to the different graphite content of the pencil lead [176,299]. In particular, it was found that the HB pencil leads (6B−12B) are more suitable for the electrodes of PB sensors (Table 7) [176]. Usually, the drawing is repeated 8–10 times to get continuity of the conductive film on paper. There is no chemical interaction between paper and graphite flakes, but graphite flakes adhere to paper due to weak van der Waals interaction with intermolecular forces. Although the PB pencil electrodes have the advantages of simple preparation, low cost and environment-friendly, they are affected by pencil types [176,299], the drawing process (repetition times) [299], and even their conductive properties are affected by bending strain [300].

Therefore, in a number of publications, another simple approach to the manufacture of paper-based electrodes has been proposed. This approach was based on the use of conductive adhesive tapes [26,28,175,178]. Adhesive copper foil and adhesive tape based on conductive polyester fibers were tested (see Figure 19d,e). The experiment showed that the electrode rigids of adhesive copper foil tapes cannot resist the continuous bending and twisting, resulting in poor wearable flexibility and even the damage of the sensors [28]. At the same time, it was found that the flexible conductive tape based on polyester fibers is helpful to solve the compatibility problem between the rigid electrodes and paper. In addition to the direct formation of electrodes, such a tape, applied to the surface of electrodes formed by various methods, significantly improves their mechanical stability when bending paper substrates. Using this approach, a multifunctional PB humidity sensor (see Figure 19f) was fabricated [28].

**Figure 20:**
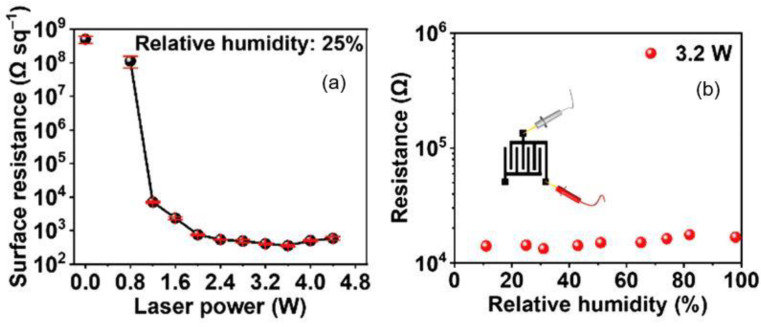
CO_2_-laser-induced direct formation of electrodes on TEMPO-oxidized cellulose paper: (**a**) surface resistance as a function of laser power used for laser irradiation, (**b**) relationship between relative humidity and electrical resistance of laser-induced electrodes prepared at laser powers of 3.2 W. Laser scan speed: 10 cm s^−1^. The resistance was measured at room temperature. Reprinted from [302]. Published 2022 by RSC as open access.

**Figure 21:**
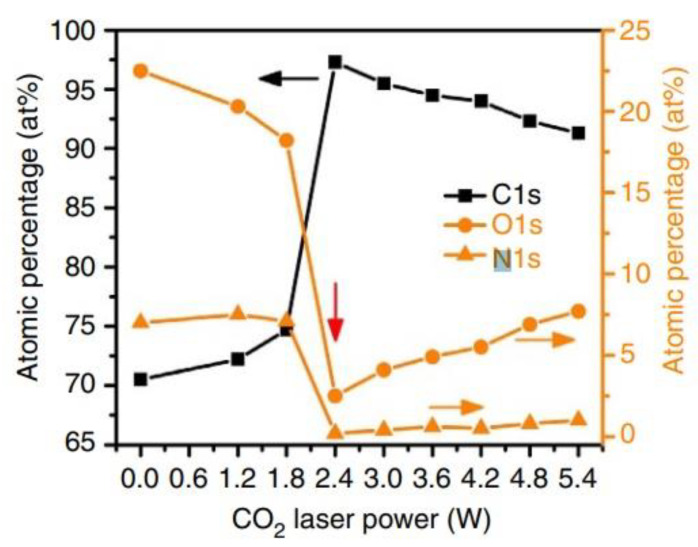
Characterizations of laser induced graphene (LIG) prepared with different laser powers. Atomic percentages of carbon, oxygen and nitrogen as a function of laser power. These values are obtained from high-resolution XPS. The threshold power is 2.4 W, at which conversion from PI to LIG occurs. Reprinted from [303]. Published 2014 by Natio Portfolio as open access.

Some developers believe that it is not economically feasible to use noble metals in paper sensors, as they are expensive and non-renewable ones. To solve this problem, a method was proposed for forming conductive electrodes using the direct CO_2_-laser writing of electrodes onto TEMPO-oxidized cellulose paper [302]. As it is seen in Figure 20, while the original TEMPO oxidized cellulose paper had a high surface resistance of approximately 10^9^ Ω sq^−1^, the laser-irradiated area had a surface resistance of 10^8^–10^2^ Ω sq^−1^, depending on the laser power. The CO_2_-laser-induced carbonization of polymeric materials can be attributed to the generation of thermal energy due to the photothermal effect derived from their lattice vibrations [303]. When subjected to CO_2_-laser irradiation, the polymeric material reaches a high temperature, which causes chemical bonds such as C–O and C=O within the polymer to break and rearrange to form a graphitic structure (Figure 21). This approach allowed to realize an all-cellulose-derived humidity sensor. The TEMPO-oxidized cellulose paper with sodium carboxylate groups provided a satisfactory humidity-sensing performance [302]. Zhu et al. [302] and Lin et al. [303] believe that the CO_2_-laser-irradiation process is extremely attractive for industrial use because it can be entirely performed in ambient air without any reagents.

If electrodes are required that are conductive over the whole area of the paper, then commercially available carbon paper can be used for this purpose, or metals, such as Au, can be deposited on the whole area of the paper. Another possibility to achieve this goal is to pyrolyze the paper by heating it to ~900–1000 °C in an inert atmosphere. However, the resulting material is rather brittle [304]. It was shown that pyrolysis can also be carried out locally using a laser [165]. It is also possible to make conductive paper by mixing conductive materials with cellulose or by coating the surface of the paper with conductive polymers such as PEDOT/PSS [74,279,305].

Additional information regarding the formation of electrodes on paper can be found in the reviews by Cummins and Desmulliez [278], Hoeng et al. [82], Liu et al. [71], Yao et al. [75,280], Zhang et al. [70], Noviana et al. [306], and Mazurkiewicz et al. [304]. Cummins and Desmulliez [279] considered inkjet printing of conductive materials. Yao et al. [280] summarized the advances of paper-based electrodes for flexible energy storage devices. Mazurkiewicz et al. [304] analyzed the features of the formation of electrodes for paper-based electrochemical sensors. Hoeng et al. [82] and Zhang et al. [70] analyzed devices based on nanopaper and features of using printing technology for their manufacture.

## 6. Summary

Thus, we considered the possibilities of using paper for the development of humidity sensors and showed that paper is indeed a promising material for these applications, not only as a substrate, but also as a humidity-sensitive material. The review showed that all types of humidity sensors, including capacitive, resistive and impedance, can be developed based on the paper, and all types of humidity-sensitive materials such as carbon-based materials, solid state materials and polymers, can be used in these sensors. At that PB humidity sensors can be highly efficient in humidity monitoring. However, to achieve maximum sensitivity, the properties of the humidity sensitive materials used in PB sensors must be optimized. The analysis also showed that printing technologies are the most suitable for the formation of both a sensitive layer and electrodes on a paper substrate. In the second part of this review, “Humidity sensors performances”, we will already consider the specific approaches used to manufacture such paper-based humidity sensors. In conclusion, we will analyze the problems that need to be solved in order to promote paper-based humidity sensors on the sensor market and discuss the current trends in their development.

## Figures and Tables

**Table 1:** Microstructure and characteristics of some cellulose papers.

Paper Type	SEM Images or Photograph	Remarks
Whatman #1 filter paper (Chromatographic paper). Reprinted with permission from [99]. Copyright 2015: Elsevier.	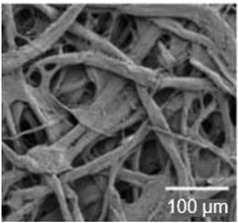	Smooth surface; almost consists of pure cellulose; no other impurities; superior aperture; the thickness and structure are relatively uniform.
Nanocellulose paper. Reprinted with permission from [100]. Copyright 2016: American Chemical Society.	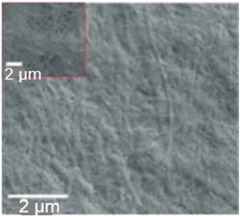	Dense structure; glaze surface; high grade of transparency; good hydrophobicity.
Anisotropic, transparent paper. Inside the anisotropic transparent film showing aligned microfibers. Reprinted with permission from [101]. Copyright 2017: Wiley-VCH.	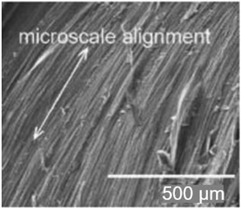	Anisotropy; high transparency; high mechanical tensile strength.
Nitrocellulose paper. Reprinted with permission from [102]. Copyright 2020: Elsevier B.V.	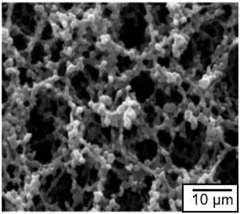	Excellent biocompatibility; unique physical and chemical properties; the ability to immobilize various biomolecules.
Transparent microcrystalline cellulose/polyvinyl alcohol paper. Reprinted with permission from [103]. Copyright 2020: American Chemical Society.	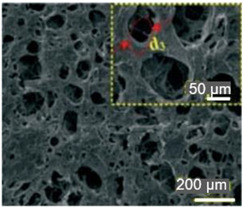	Adjustable aperture; high light transmittance (>95%); good mechanical properties; excellent biocompatibility

**Table 2:** Comparison of cellulose nanopaper, traditional paper, and plastic.

Characteristics	Cellulose Nanopaper	Traditional Paper	Plastic Paper
Surface roughness (nm)	5	5000–10,000	5
Porosity (%)	20–40	50	0
Pore size (nm)	10–50	3000	0
Optical transparency at 550 nm (%)	90	20	90
Max loading stress (MPa)	200–400	6	50
Coefficient of thermal expansion (CTE) (ppm K^−1^)	12–28.5	28–40	20–100
Printability	Good	Excellent	Poor
Young modulus (GPa)	7.4–14	0.5	2–2.7
Bending radius (mm)	1	1	5
Renewability	High	High	Low

Source: Reprinted with permission from [104]. Copyright 2013: American Chemical Society.

**Table 3:** Comparison of properties and cost for classical printed electronics substrates (plastic and conventional paper) and cellulose nanopaper.

Parameter/Material	PET	Conventional Paper	Cellulose Nanopaper (Based NFCs)	Cellulose Nanopaper (Based TEMPOCNFs)
Surface Roughness (nm)	≈0.5–2	≈10	≈2–40	≈0.2–0.5
Young modulus (GPa)	≈2–2.5	≈2	≈10	≈10–13
Coefficient of thermal expansion (ppm K^−1^)	50–200	—	≈8–13	≈7.2–7.9
Paper Transparency	Yes	No	Yes	Yes
Price (€ m^−2^)	4–6	6–7	15–200	200–500
Scale-up	Yes	Yes	No	No

Source: Reprinted with permission from [82]. Copyright 2016: Royal Society of Chemistry.

**Table 4:** Comparison of cellulose paper with traditional materials as substrates.

Property			Material	
	Glass	Silicon	Polydimethylsiloxane (PDMS)	Cellulose Paper
Surface profile	Very low	Very low	Very low	Moderate
Flexibility	No	No	Yes	Yes
Structure	Solid	Solid	Solid, gas permeable	Fibrous
Surface-to-volume ration	Low	Low	Low	High
Fluid flow	Forced	Forced	Forced	Capillary action
Sensitivity to moisture	No	No	No	Yes
Biocompatibility	Yes	Yes	Yes	Yes
Disposability	No	No	No	Yes
Biodegradability	No	No	To some extent	Yes
High-throughput fabrication	Yes	Yes	No	Yes
Functionalization	Difficult	Moderate	Difficult	Easy
Spatial resolution	High	Very high	High	Low to moderate
Homogeneity of the material	Yes	Yes	Yes	No
Price	Moderate	High	Moderate	Low
Initial investment	Moderate	High	Moderate	Low

Source: Reprinted with permission from [108]. Copyright 2013; Springer.

**Table 5:** Possible mechanisms for moisture transport through paper.

Transport Mechanism	Transported Phase	Place of Transport	Transport Coefficient	
			Dependence on Temperature	Dependence on RH
Gas diffusion	Gas phase	The pores	Proportional to T^1.76^	Independent (apart from the effects of swelling)
Knudsen diffusion	Gas phase	Pores with diameters less than 10 nm	Proportional to T^1/2^	Independent (apart from the effects of swelling)
Surface diffusion	Adsorbed phase	Surface of the fibers		Increases with increasing RH
Bulk-solid diffusion	Absorbed phase	Within the fibers		Increases with increasing RH
Capillary transport	Condensed phase	The pores		Only when pores are filled with water

Source: Reprinted with permission from [242]. Copyright 1993: Taylor and Francis.

**Table 6:** Advantages and disadvantages of the main fabrication techniques for paper-based sensors.

Procedure	Advantages	Disadvantages
Wax printing	Low-cost, easy fabrication, short fabrication time	Low resolution, unstable upon heating
Photolithography	High resolution, suitable for large-scale production	Expensive and sophisticated equipment, expensive reagents, instability against bending or folding
Inkjet printing	Efficient, reduced cross-contamination, rapid fabrication, high resolution	Expensive ink printer, different inks are needed, the heating is required
Screen printing	Low cost and simple operation	Low resolution, rough microfluidic channel walls, different molds need to be customized according to needs
Laser cutting	Simple	Specialized equipment is needed
Plasma treatment	Use of cheap plate-making agent, low material cost	Plasma reactor is needed, need to customize the mold according to needs
Polydimethyl-Siloxane (PDMS) printing	Low-cost, flexible	Low resolution, sophisticated equipment for fabrication of molds
Knife plotting	No hydrophobic modification materials are required	X–Y knife plotter is required, possibility of tearing paper during cutting
Hot Embossing	Short fabrication time, efficient	Specialized equipment
Hydrophobic silanization	Low-cost, rapid fabrication	Limitation with simple designs
Origami and Kirigami	Intricate and innovative designs, simple fabrication	

Source: Data extracted from [75,144].

**Table 7:** The bulk conductance of pencil leads from 1B to 12B and square resistances of pencil trace on the printing paper. The preparation of pencil trace is analogous with the fabrication of electrodes.

Pencil	B	2B	3B	4B	5B	6B	8B	10B	12B
Square resistance (Ω)	8717	6776	4163	2168	509	368	332	410	643

Source: Reprinted with permission from [176]. Copyright 2017: ACS.

## Data Availability

Not applicable.
